# Combinatorial characterization of metastable luminous silver cations

**DOI:** 10.1038/s41598-024-55014-8

**Published:** 2024-02-26

**Authors:** Hirokazu Masai, Masanori Koshimizu, Hiroki Kawamoto, Hiroyuki Setoyama, Yohei Onodera, Kazutaka Ikeda, Shingo Maruyama, Naoki Haruta, Tohru Sato, Yuji Matsumoto, Chika Takahashi, Teruyasu Mizoguchi

**Affiliations:** 1 Department of Materials and Chemistry, National Institute of Advanced Industrial Science and Technology, 1-8-31 Midorigaoka, Ikeda, Osaka 563-8577 Japan; 2 Research Institute of Electronics, Shizuoka University, 3-5-1 Johoku, Naka-Ku, Hamamatsu, Shizuoka, 432-8011 Japan; 3 Department of Applied Chemistry, Tohoku University, 6-6-07 Aoba, Sendai, Miyagi 980-8579 Japan; 4 Kyushu Synchrotron Light Research Center (SAGA Light Source), 8-7 Yayoigaoka, Tosu, Saga 841-0005 Japan; 5 Institute for Integrated Radiation and Nuclear Science, Kyoto University, 2-1010 Asashiro-Nishi, Kumatori-Cho, Sennan-Gun, Osaka 590-0494 Japan; 6 Institute of Materials Structure Science, High Energy Accelerator Research Organization (KEK), 203-1 Shirakata, Tokai-Mura, Naka-Gun, Ibaraki 319-1106 Japan; 7 Fukui Institute for Fundamental Chemistry, Kyoto University, Takano-Nishihiraki-Cho 34-4, Sakyo-Ku, Kyoto, 606-8103 Japan; 8 Department of Molecular Engineering, Graduate School of Engineering, Kyoto University, Nishikyo-Ku, Kyoto, 615-8510 Japan; 9 Institute of Industrial Science, The University of Tokyo, 4-6-1, Komaba, Meguro, Tokyo, 153-8505 Japan; 10 Present Address: Center for Basic Research on Materials, National Institute for Materials Science, 1-2-1 Sengen, Tsukuba, Ibaraki 305-0047 Japan

**Keywords:** Materials science, Optics and photonics

## Abstract

Thermodynamically metastable glasses that can contain metastable species are important functional materials. X-ray absorption near-edge structure (XANES) spectroscopy is an effective technique for determining the valence states of cations, especially for the doping element in phosphors. Herein, we first confirm the valence change of silver cations from monovalent to trivalent in aluminophosphate glasses by X-ray irradiation using a combination of Ag L_3_-edge XANES, electron spin resonance, and simulated XANES spectra based on first-principles calculations. The absorption edge of the experimental and simulated XANES spectra demonstrate the spectral features of Ag(III), confirming that AgO exists as Ag(I)Ag(III)O_2_. A part of Ag(I) changes to Ag(III) by X-ray irradiation, and the generation of Ag(III) is saturated after high irradiation doses, in good agreement with conventional radiophotoluminescence (RPL) behaviour. The structural modelling based on a combination of quantum beam analysis suggests that the local coordination of Ag cations is similar to that of Ag(III), which is confirmed by density functional theory calculations. This demonstration of Ag(III) in glass overturns the conventional understanding of the RPL mechanism of silver cations, redefining the science of silver-related materials.

## Introduction

The valence states of cations dominate material parameters including electrical, optical, and mechanical properties. Because precise material design requires tailoring the valence state as well as the connectivity of the elements, examination of the valence state is an integral part of material science. A small number of minor elements can sometimes be critical for performance improvement. In activator-doped materials, the valence states of activators are responsible for their luminescence properties. In these cases, X-ray absorption fine structure (XAFS) spectroscopy can be used to determine the local coordination^1–13^. However, it is not straightforward to assign the local coordination state of activators in amorphous materials because of a lack of periodic structures. Although conventional XAFS measurements have been performed using K-edge absorption, K-edge analysis, which can cover a broader energy range than other absorption edges, is sometimes ineffective for heavier cations in amorphous materials because of the heavy-atom effect and ambiguity of the s-p transition in K-edge absorption^14,15^. In such cases, L-edge X-ray absorption near-edge structure (XANES) spectroscopy, rather than K-edge spectroscopy, can be used to determine the valence state of elements more accurately.

One unique phenomenon of activators in glasses is that unstable crystalline phases or metastable valences of cations can exist in the matrix under ambient conditions, i.e., the so-called “confinement effect of glass”. The generation of divalent rare-earth cations is a typical example of metastable species in a glass matrix^16,17^. Such metastable valences can be generated by external stimulation such as a laser or radiation^16^. However, radiation-induced valence changes sometimes induce the generation of another emission centre, i.e., radiophotoluminescence (RPL)^18–28^. RPL is a dosimetry technique in which stored energy can be detected using stimulated light wave irradiation. The generation of metastable activators is important for RPL; however, only a limited number of materials exhibits RPL behaviour. Herein, we focus on silver-doped phosphate glasses, which is a material that exhibits RPL behaviour.

Commercially available dosimeters comprised of Ag-doped oxide glass badges are used for personal monitoring using RPL behaviour. In the RPL mechanism reported in the literature for silver-doped phosphate glasses^18–20^, certain Ag^+^ cations in a host glass change to Ag^0^ by trapping an electron by radiation, whereas other Ag^+^ cations change to Ag^2+^ through a combination of holes. The valence change is speculated from spectroscopic data; however, direct valence observation using XAFS has not been reported to date. Recently, our group reported a valence change during X-ray irradiation of Ag-doped phosphate glasses using Ag L-edge XANES spectroscopy, in which a new peak generated by X-ray irradiation was observed at a low absorption energy^15^. It should be noted that the peak energy of the white line of Ag shifts toward a lower energy as the oxidation state increases, in contrast to those of conventional elements. Although we propose the possibility that a higher valence state of silver species is formed based on Ag L_3_-edge XANES spectra by comparison with an AgO reference^15^, there is insufficient evidence on the origin of the generated Ag species. Therefore, a comparison with other silver reference materials is needed to elucidate the mechanism of the valence change of Ag during X-ray irradiation. In addition, we examined the relationship between the generated peaks and conventional RPL behaviour.

In this study, we focussed on silver-doped phosphate glass, which exhibits RPL behaviour. We initially discuss the relationship between RPL properties and X-ray-induced Ag species in aluminophosphate-based FD-7 glass, commercially used as glass badges for personal monitoring, based on Ag L_3_-edge XANES analysis. We then attempted to confirm the presence of higher-valence silver species in the glasses by comparison with silver oxide clathrate. We obtain theoretical proof based on first-principles calculations of the absorption edge shift in XANES and demonstrate that AgO exists as Ag(I)Ag(III)O_2_. Moreover, using density functional theory (DFT) calculations, we demonstrate the existence of metastable trivalent Ag in glass. Both experimental and theoretical results support the existence of trivalent Ag in glass, contradicting the conventional understanding of RPL behaviour^18–28^.

## Results

### Relationship between XANES peak of generated Ag species and RPL behaviour

The FD-7 glass, the chemical composition of which is shown in Table S1, has been used as glass badges for personal monitoring provided by the Chiyoda Technol Corporation. The physical and structural data of FD-7 glass are shown in Fig. S1. The conventional understanding of the RPL behaviour of Ag-doped glasses^18–28^ is that both Ag^0^ and Ag^2+^ are generated from Ag^+^ by irradiation, and the RPL behaviour is mainly affected by Ag^2+^. For L-edge XANES analysis, transitions of the L_3_- and L_2_-edge, arising from the 2p_3/2_ → 4d_5/2_ and 2p_1/2_ → 4d_3/2_ transitions, respectively, are suitably compared with the L_1_-edge (from 2 s) owing to the dipole-allowed transition. In addition, considering degeneracy, the L_3_-edge is the most preferable for discussion of the change in the white line (Fig. S2). In a previous study, which was the first examination of Ag L_3_-XANES spectra for Ag-doped phosphate glasses, an absorption peak appeared at 3.349 keV for prepared FD-7 glass as a function of the electrical current in the storage ring multiplied by time^15^. Herein, we estimated the X-ray dose on the sample considering the experimental conditions at the Kyushu Synchrotron Light Research Center (Saga, Japan). We estimated the X-ray photon rate at the sample position to be 3.0 × 10^10^ photons s^−1^ for a spot size of 0.1 × 0.6 cm^2^ (Fig. S3) and ring current of 170 mA (Fig. S4). In addition, the X-ray attenuation length for the composition and density of the sample (2.6 g cm^−3^) was estimated to be 9.0 μm at the sample surface on average for the X-ray energy range employed in this study. Considering the estimated energy flux at the surface of 2.9 × 10^−4^ J s^−1^ cm^−3^, the dose rate at the surface is estimated to be 0.11 Gy s^−1^. Thus, the X-ray dose corresponding to 1 mA∙h is estimated to be 2.4 Gy.

**Figure 1:**
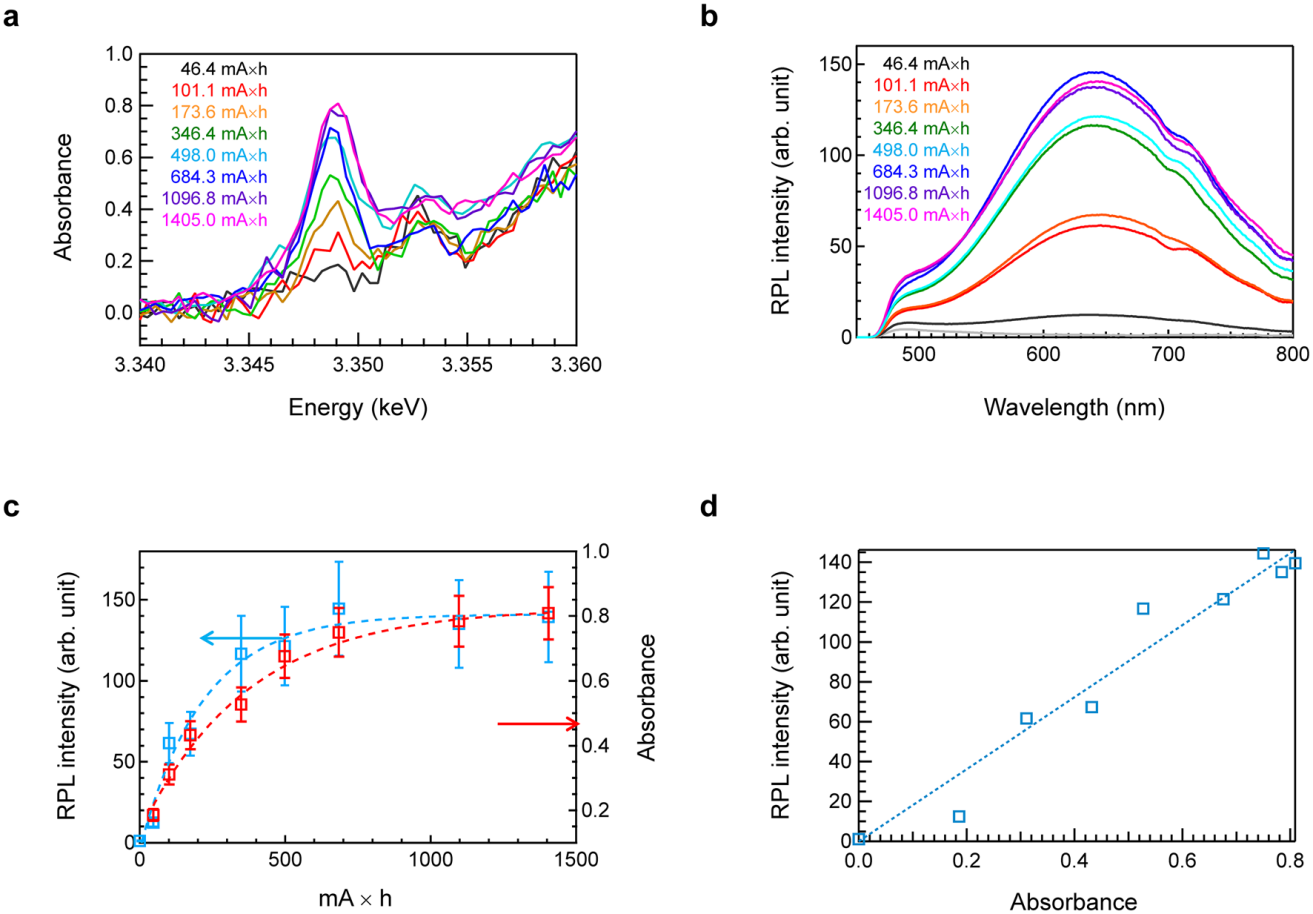
Saturation behaviour of FD-7 glass after high irradiation doses. (**a**) Ag L_3_-XANES spectra of FD-7 glass after different irradiation doses. (**b**) RPL intensity of FD-7 glass after each Ag-L_3_-XANES measurement. (**c**) Relative intensities of RPL and X-ray induced absorption at 3.349 keV in Ag L_3_-edge XANES as a function of irradiation dose. (**d**) Comparison of the relative intensities of RPL and X-ray induced absorption of Ag.

Figure 1a shows the Ag L_3_-XANES spectra of the commercial FD-7 glass after different irradiation doses. Similar to the prepared FD-7 glass^15^, the peak intensity at 3.349 keV increased with increasing irradiation dose and seemed to be saturated after a long irradiation time (Fig. S5). After a long irradiation time, the generation of brown colour center could be observed by the naked eye. Although the beam size for the XANES measurement (Fig. S3) was not suitable for the measurement of optical absorption spectra, we confirmed an increase in the absorption band in the UV region by X-ray irradiation using another experimental setup (Fig. S6). The intensity of the absorption attributable to Ag^+^ at 3.353 keV does not significantly decrease even after a long irradiation duration. If all Ag^+^ cations are changed to other valence states by X-ray irradiation, a significant decrease in the Ag^+^ peak after long-duration irradiation should be observed. However, such a change was not observed when differential XANES spectra were obtained by subtracting the first scanning spectrum (Fig. S7). Therefore, the results clearly confirm that not all Ag^+^ species change upon X-ray irradiation and that a very small amount of Ag^+^ is involved in the valence change. Figure 1b shows the RPL intensity of FD-7 glass after each Ag-L_3_-XANES measurement. The excitation wavelength is 3.91 eV (317 nm). The RPL intensity of the FD-7 glass appears to be saturated after a long irradiation time. To compare both trends, we plotted both peak intensities to confirm the saturation behaviour. Figure 1c shows the relative intensities of the RPL and X-ray-induced absorption at 3.349 keV in Ag L_3_-edge XANES as a function of the electrical current multiplied by time. The intensity changes in both measurements appear to be saturated at approximately 800 mA h, corresponding to an irradiation dose of ~ 2 kGy. This dose dependence is consistent with the results of a previous study^27^. The similarity between the two parameters can be understood from a direct comparison between the relative intensities of the RPL and generated X-ray-induced absorption, as shown in Fig. 1d. Because there is good agreement between them, we conclude that the peak generated by X-ray irradiation is the origin of the RPL behaviour.

### Reversibility of Ag species by X-ray irradiation

**Figure 2:**
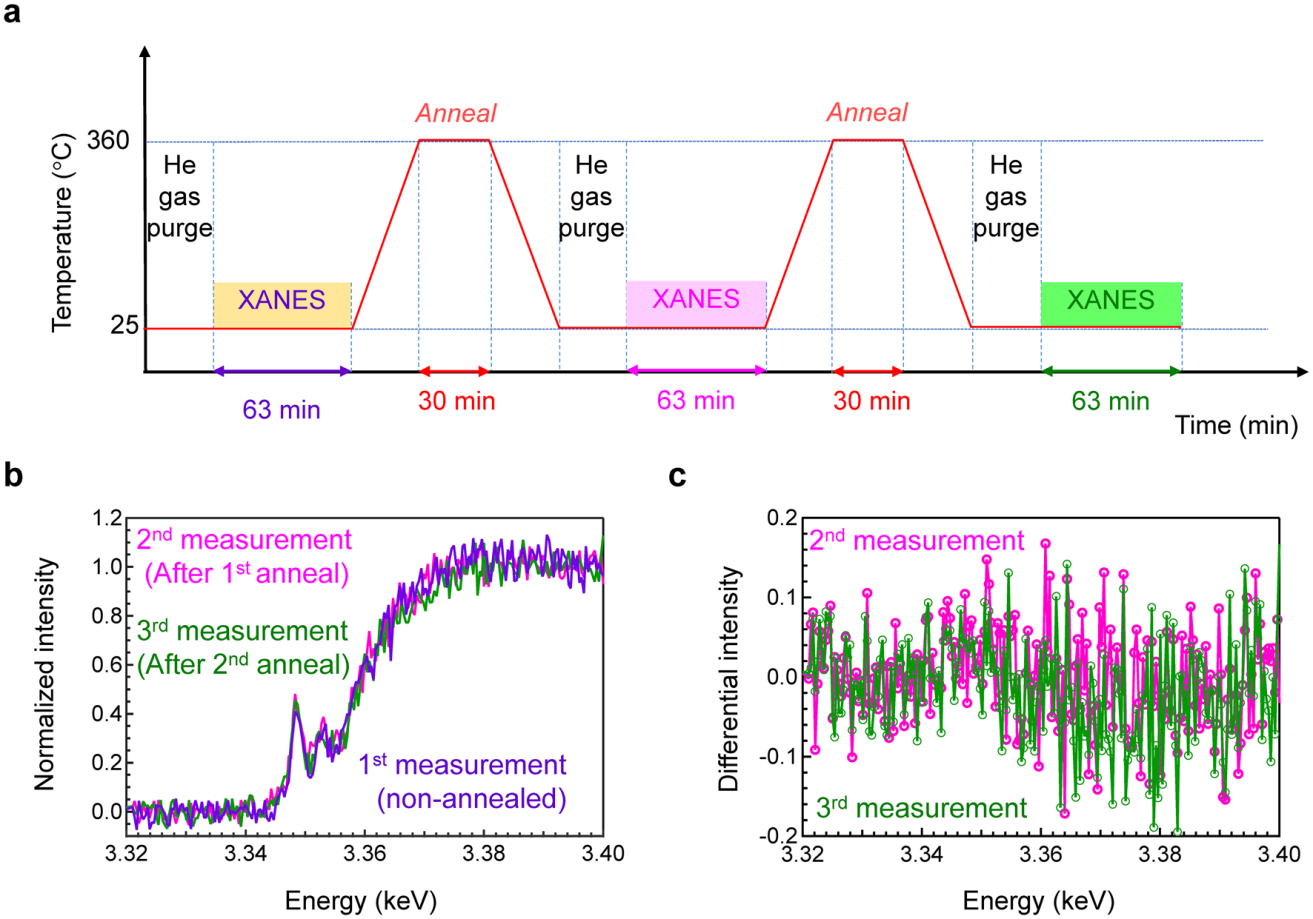
Reversibility of X-ray-induced generated peak after annealing. (**a**) Schematic illustration of the used protocol for the XANES reversibility measurement. (**b**) Ag L_3_-edge XANES spectra of FD-7 glass with and without annealing. These spectra were obtained after the same irradiation doses. (**c**) Differential Ag L_3_-edge XANES spectra of FD-7 glass after the same irradiation doses, obtained by subtracting the spectrum of non-annealed FD-7 glass.

According to the conventional understanding of the RPL behaviour of Ag-doped phosphate glass, the X-ray-induced valence change of Ag cations can be recovered by thermal annealing. The glass transition temperature of FD-7 glass is 450 °C^15^, and reportedly, thermal annealing at 360 °C is sufficient to erase defects^28^. Based on previous reports, the reversibility of the Ag valence change was examined using the same glass sample. A schematic illustration of the used protocol for the XANES reversibility measurements with an annealing process is shown in Fig. 2a. The detailed measurements are presented in experimental section. The process of X-ray irradiation followed by thermal annealing was performed twice to confirm the reversibility. The resulting XANES spectra are depicted in Fig. 2b. The differential Ag L_3_-edge XANES spectra of FD-7 glass after each irradiation cycle is shown in Fig. 2c, obtained by subtracting the spectrum of the non-annealed FD-7 glass (the initial state). The differences between these spectra were within the range of the error bars, indicating that the generated defects disappeared after thermal annealing. Therefore, we have confirmed that the generated Ag species, which correlates with RPL behaviour, are thermally annealable (reversible), consistent with the conventional RPL mechanism.

### Valence estimation of silver by comparison with silver oxide clathrate Ag_7_O_8_NO_3_

**Figure 3:**
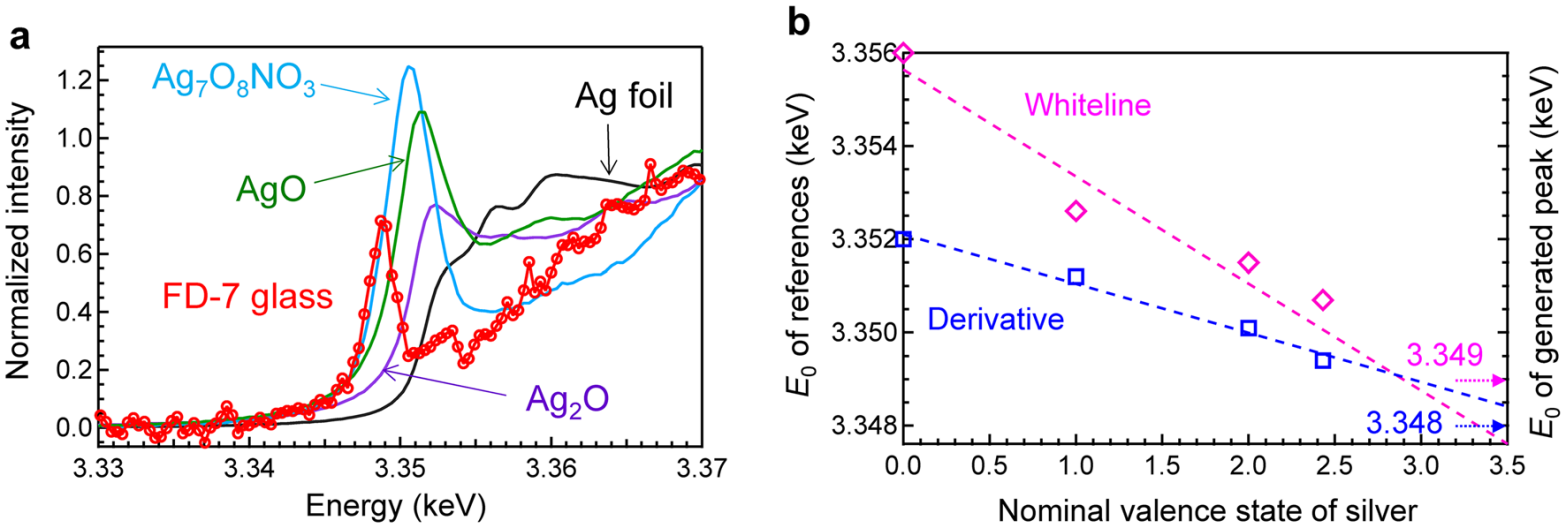
Comparison of Ag L_3_-XANES spectra. (**a**) Ag L_3_-edge XANES spectrum of FD-7 glass after 1,405 mA∙h, along with those of Ag foil, Ag_2_O, AgO, and Ag_7_O_8_NO_3_. (**b**) Absorption edge energy *E*_0_ of reference materials as a function of the valence state of Ag. The *E*_0_ values of the generated peak defined by the zero of the second derivative and the peak of the white line are depicted at the right axis.

Recently, Ag L_3_-edge XANES analysis was reported to be effective for evaluating the valence state of silver^15^. The peak energy of the white line shifts toward lower energies as the oxidation state increases, which is opposite to the behaviour of conventional elements. This different spectral shift is caused by the generation of empty states at the conduction band bottom in the higher oxidization state of Ag. In addition, additional absorption bands, whose peak intensities increased with increasing irradiation dose, were generated at lower absorption energies during the measurement. However, from the trend in the absorption energies of the Ag species, we could only assign peaks to Ag valences higher than 2 in the previous report^15^. To clarify the valence state of the generated peaks, we measured the Ag L_3_-edge XANES spectrum of silver oxide clathrate Ag_7_O_8_NO_3_^29^. The nominal valence state of silver in Ag_7_O_8_NO_3_ was calculated to be approximately 2.43, which is higher than that of the reference AgO. Figure 3a shows the Ag L_3_-edge XANES spectrum of commercial FD-7 glass after 1,405 mA∙h irradiation, along with those of Ag foil, Ag_2_O, AgO, and Ag_7_O_8_NO_3_. The peak of Ag_7_O_8_NO_3_ is located at an absorption energy lower than that of AgO, and its peak height is higher than that of AgO. Therefore, the XANES result of Ag_7_O_8_NO_3_ is consistent with the tendency of the Ag L_3_-edge XANES spectra, i.e., a higher valence state of Ag results in a lower absorption edge energy. The absorption energy of the peak generated for the FD-7 glass is located at a lower absorption energy than that of Ag_7_O_8_NO_3_. To estimate the valence state of silver, we plotted the absorption edge energy *E*_0_ of the reference materials as a function of the valence state of Ag, as shown in Fig. 3b. Because the value of *E*_0_ is affected by its definition^14^, we used two definitions for FD-7 glass: zero of the second derivative and the peak of the white line, resulting *E*_0_ values of 3.348 and 3.349 keV, respectively. The values of *E*_0_ are depicted with arrows on the right axis of Fig. 3b. The generated peak is attributed to the Ag species possessing a valence state of approximately 3. By linear fitting of these data and extrapolation of both *E*_0_ values, we assumed that the generated Ag cations were trivalent. The validity of this estimation is discussed based on the simulated XANES spectra.

### Comparison of Ag species detected by electron spin resonance (ESR) and XANES

**Figure 4:**
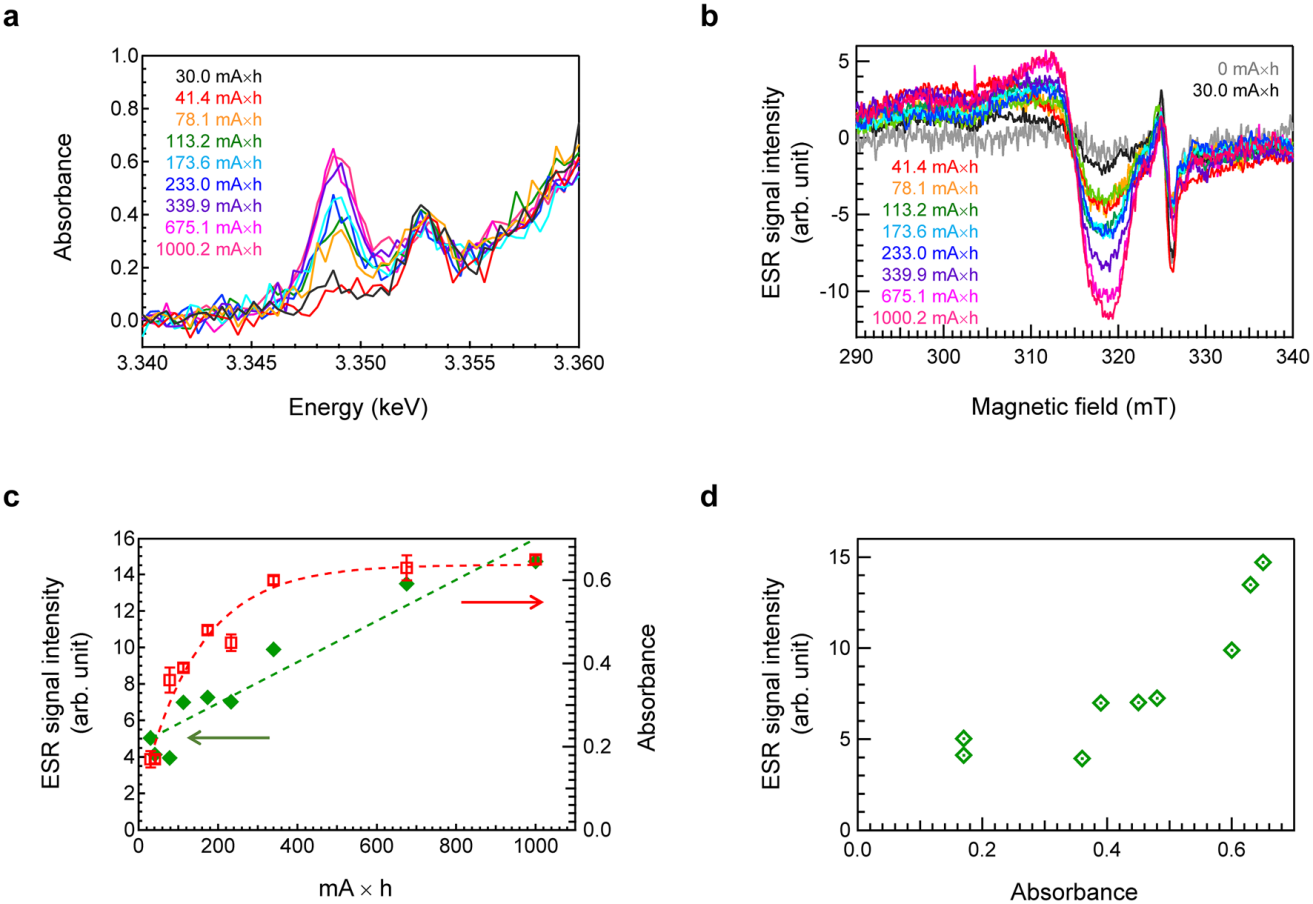
Irradiation dose dependence of ESR and L_3_-edge XANES spectra. (**a**) Ag L_3_-XANES spectra of FD-7 glass after different irradiation doses. (**b**) ESR spectra of FD-7 glass after different irradiation doses, which were measured at − 170 °C. (**c**) Relative intensities of X-ray induced absorption at 3.349 keV in Ag L_3_-edge XANES and ESR intensity as a function of irradiation dose. (**d**) Comparison between the ESR intensities and generated absorbance in Ag L_3_-edge XANES spectra by X-ray irradiation.

One RPL-active Ag species in the conventional understanding of RPL is thought to be Ag^2+^, whose electron configuration of 4d^9^ is detectable by ESR. In contrast, Ag^3+^, which has an electron configuration of 4d^8^, is considered ESR-silent. Because the detectable minimum concentrations for ESR and XANES are different, a comparison of the irradiation dose dependence of ESR and XANES is important to conclude the generated Ag species. To obtain an accurate ESR signal of FD-7 glass, it was cut with a width of 2 mm, and ESR measurements of the bulk glasses were performed at − 170 °C. Figure 4a shows the Ag L_3_-XANES spectra of FD-7 glass after different irradiation doses. Figure 4b shows the ESR spectra of the FD-7 glasses after XANES measurements at different irradiation doses. The sharp signal at a magnetic field of 325 mT can be attributed to the oxygen hole centre with a *g* value of 2.007^30,31^. Considering the ESR spectrum of the SiO_2_ tube used for the measurements (Fig. S8), it is clear that the sharp peak is not from FD-7 glass but from the SiO_2_ tube. Figure 4c shows the relative intensities of X-ray-induced absorption at 3.349 keV in Ag L_3_-edge XANES and ESR intensity as a function of the irradiation dose. The ESR intensity was calculated from the intensity difference (g = 2.072) above and below the peak at 314 mT, after subtracting the ESR spectrum of the non-irradiated FD-7 glass (Fig. S8). The dose dependence of the ESR signal was almost linear. In contrast, a saturation tendency with respect to the irradiation dose is observed for XANES peak intensity, which is also observed in Fig. 1a. The difference in dose dependence is also understood by comparing these two intensity changes. Figure 4d shows a comparison between the ESR intensities and generated absorbance in the Ag L_3_-edge XANES spectra by X-ray irradiation. The difference in the trend with respect to irradiation dose suggests that the origins of ESR-active silver (Ag^2+^) are different from the Ag species observed by XANES. It should be noted that the concentration of Ag^2+^ species was much lower than that of Ag^3+^ species detectable by Ag XANES. Recently, we demonstrated that less than 0.1% of Ag^+^ was converted to Ag^2+^ by radiation. Therefore, it is natural that both Ag^2+^ and Ag^3+^ exist at different concentrations in FD-7 glass.

### Ag-coordination states in structural model of FD-7 glass by RMC modelling

Reliable structures of amorphous materials can be described by reverse Monte Carlo (RMC) modelling based on a combination of experimental datasets, such as X-ray diffraction (XRD) and neutron diffraction, magic angle spinning nuclear magnetic resonance (MAS NMR), and extended XAFS (EXAFS)^32–34^. Because the raw data of Ag K-edge EXAFS is not suitable for fitting because of noise of signal due to the low concentration, back-Fourier transformed EXAFS data was used (Fig. S9). Therefore, we performed RMC modelling using XRD, neutron diffraction, ^31^P and ^27^Al MAS NMR, and EXAFS data to identify the atomic configuration of FD-7 glass.

**Figure 5:**
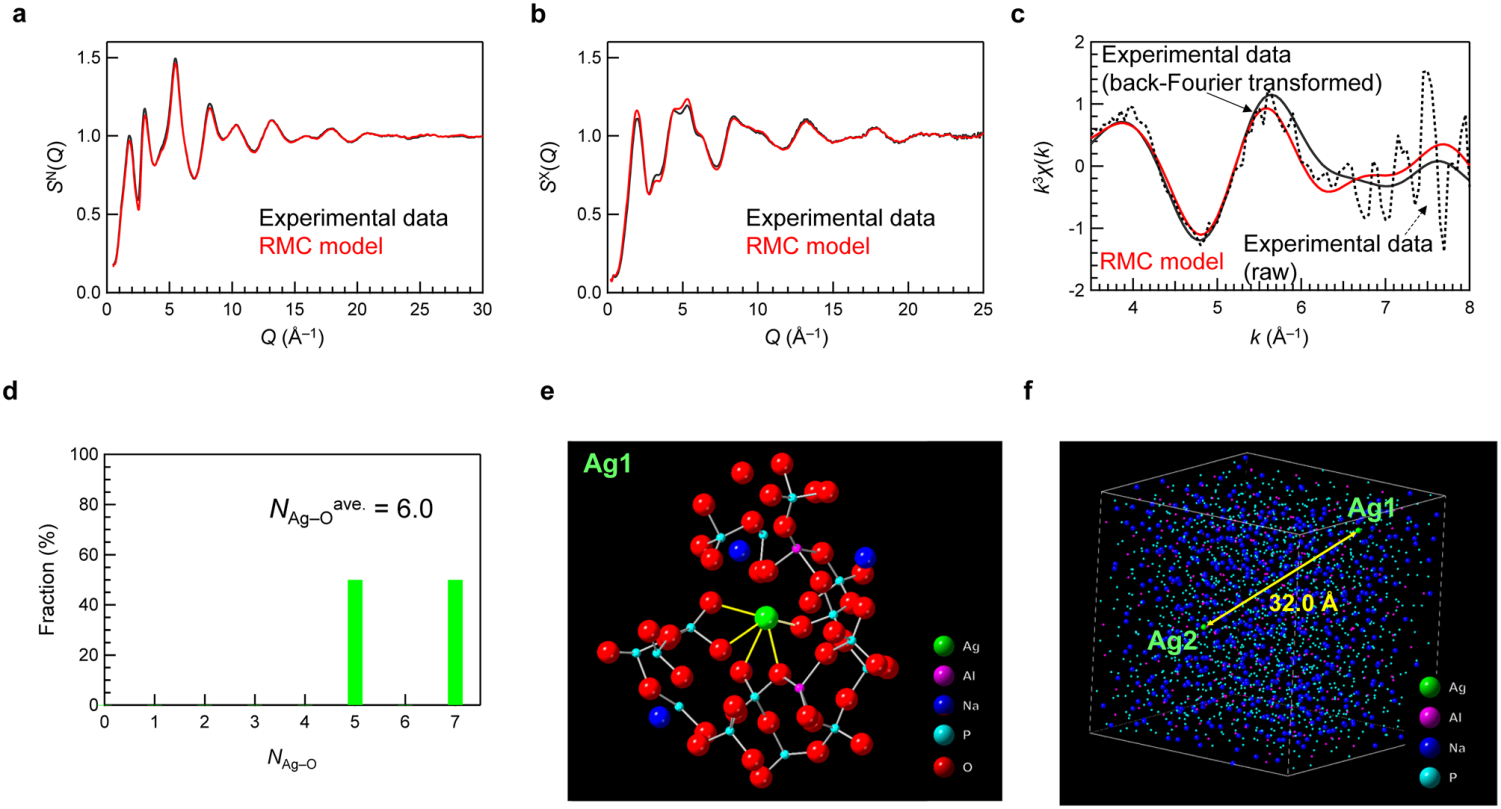
Structural modelling of Ag-doped phosphate glass based on a combination of experimental data. (**a**) Neutron total structure factor *S*^N^(*Q*), (**b**) X-ray total structure factor *S*^X^(*Q*), and (**c**) Ag K-edge EXAFS spectra of FD-7 glass. The black and red lines indicate the experimental (raw experimental data, solid line; back-Fourier transformed experimental data, dotted line) and RMC modelling results, respectively. (**d**) The coordination number distribution of oxygens around Ag. (**e**) Schematic of atomic configurations in the vicinity of Ag cations. Green: silver, purple: aluminium, blue: sodium, light blue: phosphorus, and red: oxygen. The Ag–O bonds are highlighted in yellow. (**f**) Cation distributions in FD-7 glass obtained from the RMC modelling.

Aluminium cations have been reported to undergo 4-, 5-, and 6-fold coordination with oxygen in FD-7 phosphate glasses^15^. Figure 5a–c show the experimental and RMC modelled neutron total structure factor *S*^N^(*Q*), X-ray total structure factor *S*^X^(*Q*), and Ag K-edge EXAFS spectra of FD-7 glass, respectively. The details of the modelling are described in the Materials and Methods section. Note that no coordination number constraints for Ag cations were applied in the RMC modelling. There is good agreement between the RMC modelling and experimental results for FD-7 glass. The cation–oxygen coordination number distributions of Na, P, and Al in FD-7 glass are shown in Fig. S10a–c, respectively, in addition to the partial pair correlation function *g*_*ij*_(*r*) (Fig. S10d). The Ag–oxygen coordination numbers obtained from the RMC modelling are shown in Fig. 5d. With a threshold of 3.0 Å, determined from the partial-pair correlation function for Ag–O, the average coordination number of Ag after X-ray irradiation is calculated to be 6.0. Notably, this RMC-generated coordination number is similar to that of Ag^3+^. Figure 5e shows the atomistic configuration near the Ag cation in FD-7 glass. As expected, Ag cations were coordinated by oxygen atoms belonging to the Q^2^ phosphate unit. Another Ag coordination mode in FD-7 glass is shown in Fig. S11, in which Ag cations are also connected to the oxygen atoms of the P=O bond. Considering that two electrons must be captured by phosphate units, phosphate chains (Q^2^ units) exhibiting a P=O bond are favourable as a counterpart. Figure 5f shows the cation distributions in FD-7 glass obtained from the RMC modelling. The distance between two Ag cations is estimated to be 32.0 Å, suggesting that it is difficult to exchange an electron directly between two Ag^+^ cations.

The RMC-generated atomistic structure (Fig. 5e) should satisfy all experimental data. Therefore, two additional RMC simulations were performed to validate the reliability of the RMC-generated atomistic model. The first used only the X-ray and neutron structure factors (without EXAFS data). The second included coordination number constraints to force Ag cations to coordinate with two oxygen atoms, which corresponds to the local structure in Ag_2_O. In the RMC model with structural constraint for Ag_2_O coordination (*N*_Ag–O_ = 2), the coordination number distributions of oxygen atoms around Ag, Na, and Al, have been changed, as shown Fig. S12a–c, respectively. Based on the RMC simulation shown in Fig. S12d and e, a comparison between the experimental data and the three RMC models is shown in Fig. S12f. As shown in Fig. S11f, RMC modelling without the Ag–O coordination constraint is most consistent with the experimental EXAFS oscillation data, whereas RMC modelling with constraints to form two-coordinated Ag does not reproduce the EXAFS data. Therefore, the Ag coordination illustrated in Fig. 5e, which is similar to that of Ag^3+^ in Ag_2_O_3_, is a reliable local structure caused by X-ray irradiation.

### Comparison between experimental and simulated L_3_-edge XANES spectra

Generally, the absorption edge shift in XANES is used to determine the valence state of a target element. The peak energy of the white line for Ag, in contrast to that for conventional elements, shifts toward lower energy as the oxidation state increases. Therefore, we must first check the validity of this spectrum assignment. The RPL mechanism in Ag-doped glass was first reported in the 1950s^20^, and an X-ray-induced valence change from Ag^+^ to Ag^2+^ was proposed from the beginning. In contrast to the conventional understanding, our previous report indicated that the silver species generated by X-ray irradiation results in a valence state higher than two. In the present study, from the XANES results of the silver oxide clathrate, it is strongly suggested that the generated silver species are of higher valence than the traditionally considered Ag^2+^, i.e., trivalent. Because the intensity of the generated Ag species in XANES exhibits saturation behaviour very similar to that of the RPL intensity as a function of irradiation dose, and that the annealing experiment also confirmed that the Ag species is a thermally reversible species, it is speculated that Ag^3+^ is a real and key component of the RPL behaviour in Ag-doped phosphate glass. In addition, RMC-modelled Ag coordination states in FD-7 glass also suggest the existence of Ag^3+^. However, this hypothesis for Ag_2_O_3_ has not been confirmed experimentally because Ag_2_O_3_ cannot be obtained commercially because of its poor chemical stability^35^. Therefore, we attempted to simulate L-edge XANES spectra^36–38^ to confirm the presence of Ag^3+^ in FD-7 glass. In contrast to the simulation of K-edge spectroscopy, where the shape of the spectra can be easily obtained, the anisotropy of the L_3_-edge in the 2p_x_, 2p_y_, and 2p_z_ directions must be considered. The intensity of the simulated XANES spectra of each Ag species was averaged from the 2p_x_, 2p_y_, and 2p_z_ components. The detailed intensity in each direction for several Ag compounds is shown in Fig. S13. The shape of the experimental AgO spectrum could not be reproduced using AgO with the space group *Cccm*. On the other hand, it has been proposed that AgO consists of sixfold Ag(III) (A-site) and fourfold Ag(I) (B-site)^39,40^, and that the Ag site in AgO is not in a single state but is a mixture of two charge states^8,39–41^. In other words, it has been suggested that Ag(II)O is an isoelectric structure of Ag(I)Ag(III)O_2_. Considering the experimentally obtained XANES spectra, we emphasize that AgO with space group *P*2_1_/*C* is mixed-valent chemical consisting of Ag^+^ and Ag^3+^ cations. Notably, a sixfold Ag(III) site affects the intensity of the white line, which is also noticeable in Ag_2_O_3_.

**Figure 6:**
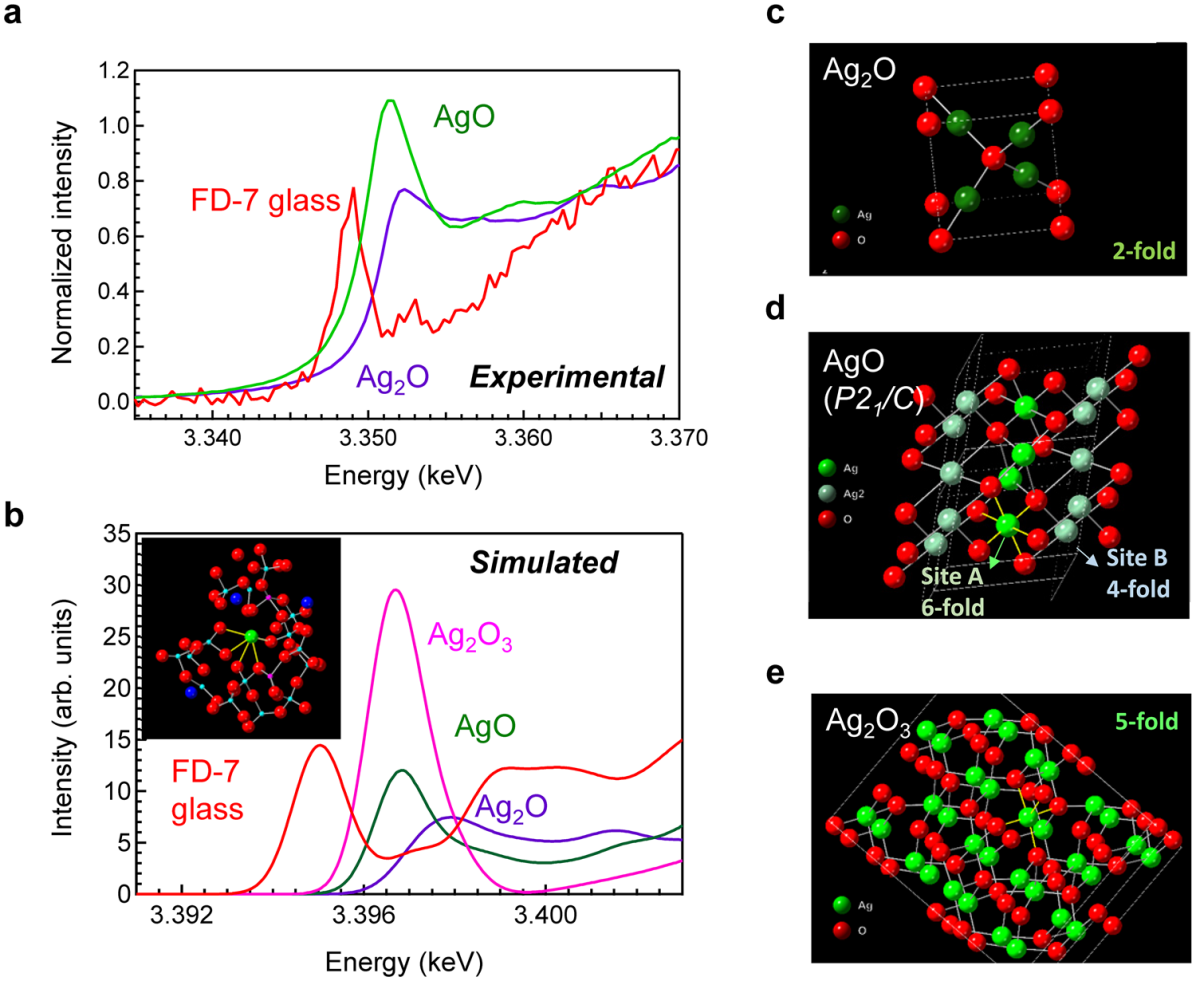
Comparison between experimental and simulated L_3_-edge XANES spectra. (**a**) Experimental Ag L_3_-XANES spectra of FD-7 glass, Ag_2_O, and AgO. (**b**) Simulated Ag L_3_-XANES spectra of FD-7 glass, Ag_2_O_3_, AgO, and Ag_2_O. Structures of (**c**) Ag_2_O, (**d**) AgO, and (**e**) Ag_2_O_3_ used for the XANES simulations.

Figure 6 shows a comparison between the experimental (Fig. 6a) and simulated (Fig. 6b) L_3_-edge XANES spectra of Ag_2_O and AgO, as well as those of Ag-doped FD-7 glass and Ag_2_O_3_ (simulated). The structures of Ag_2_O, AgO, and Ag_2_O_3_ used for the simulation of XANES spectra are shown in Fig. 6c–e, respectively. The absorption energy *E*_0_ values of these materials based on different definitions are summarized in Tables 1 and 2. A clear absorption edge shift was observed depending on the valence state of the Ag cation, and the absorption bands observed in the experimental spectra can be quantitatively assigned to those in the simulated spectra. As mentioned above, the generated absorption intensity, i.e., intensity of the white line, of Ag_2_O_3_ in the L_3_-edge XANES spectra is relatively strong compared to those of Ag_2_O and AgO. The absorption energy of the simulated spectrum for the Ag-doped FD-7 glass model is lower compared to those of Ag_2_O_3_ and AgO. Although the extent of the red-shift in the simulation is slightly greater than what is observed experimentally, the shift towards lower energy successfully reproduces the experimental trend. The simulated spectra for Ag_2_O_3_ and AgO appear at similar positions, which does not fully replicate the experimentally observed red-shift in FD7-glass. However, the present model does qualitatively capture this red-shift phenomenon. We believe that this is strong evidence of the formation of trivalent Ag in FD7-glass. It has been proposed that the absorption intensity is directly related to the de-occupation of states derived from the Ag 4d orbitals^8^. Both the experimental and simulated intensities of Ag^3+^ are stronger than those of the other Ag species, which clearly indicates that the vacant 4d orbitals affect the peak intensity. This increase in the white line intensity is more obvious in silver fluoride, as shown in Fig. S14.

**Table 1:** Experimental *E*_0_ values of Ag_2_O, AgO, Ag_7_O_8_NO_3_, and FD-7 glass derived using different definitions.

	Absorption energy *E*_0_ (keV)		
	A fraction of the edge step	The zero of the second derivative	The peak of the white line
Ag_2_O	3.3510	3.3512	3.3526
AgO	3.3493	3.3501	3.3515
Ag_7_O_8_NO_3_	3.3483	3.3494	3.3507
FD-7 glass	3.3478	3.3479	3.3489

All definitions suggest that the Ag species in FD-7 glass possess a valence state higher than 2.

**Table 2:** Simulated *E*_0_ values of Ag_2_O, AgO, and Ag_2_O_3_ based on different definitions.

	*E*_0_ (keV)		
	Absorption edge	The zero of the second derivative	Peak of the white line
Ag_2_O	3.3953	3.3970	3.3979
AgO	3.3945	3.3962	3.3968
Ag_2_O_3_	3.3945	3.3961	3.3967

In this study, we obtained clear evidence for the generation of Ag^3+^ species in Ag-doped glass by X-ray irradiation. However, there is little quantitative evidence for the change from Ag^+^ to Ag^3+^ during irradiation. As evidenced by the XANES spectra, the generated Ag^3+^ species were detectable, but a large decrease in the Ag^+^ concentration was not observed. These spectral changes are thought to be correlated with the unique characteristics of the Ag^3+^ cation, whose absorption intensity is much stronger than that of the Ag^+^ cation in the simulated XANES spectra. In other words, although the concentration of the generated Ag^3+^ species is very low, the absorption efficiency of the Ag^3+^ cation makes it easily detectable by XANES. Another important observation is that the Ag^3+^ species can survive in glass under ambient conditions. The Ag^3+^ species plays an important role in the RPL of commercially available FD-7 glass, and the fading of the luminescence properties is not very significant^42^. Considering the chemical stability of Ag_2_O_3_, which cannot be obtained commercially, the “confinement effect of glass” effectively stabilizes Ag_2_O_3_ in the glass matrix. Because the concentration of Ag^3+^ is expected to be very low, it is very difficult to detect, but a capture process of two holes in the phosphate glass network is likely to occur when Ag^+^ becomes Ag^3+^. Considering the cation ratio among P, Na, Al, and Ag^15^, it is expected that phosphate chains can capture electrons from Ag^+^ cations.

### Optical absorption and luminescence of the Ag(III) cation based on DFT calculations

**Figure 7:**
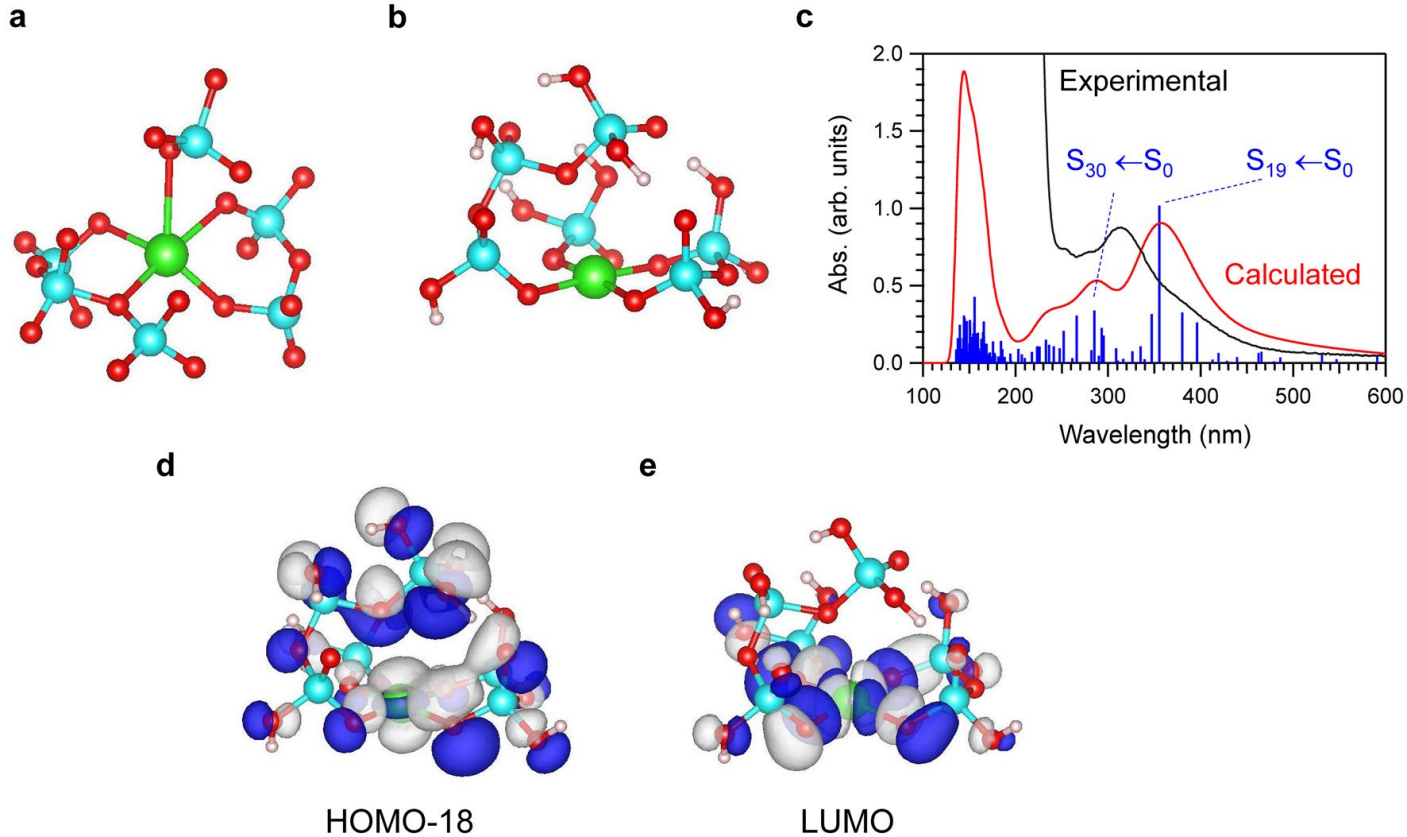
Theoretical calculation of the optical absorption spectrum of Ag in phosphate glass. (**a**) The silver(III) phosphate cluster AgP_6_O_21_ based on the RMC model. Green: silver, light blue: phosphorus, and red: oxygen. (**b**) The geometry optimized hydrogen-terminated silver(III) phosphate model AgP_6_O_21_H_11_. (**c**) The calculated absorption spectra for AgP_6_O_21_H_11_. (**d**) Illustration of HOMO-18 orbital of the silver(III) phosphate model AgP_6_O_21_H_11_. (**e**) Illustration of LUMO orbital of the silver(III) phosphate model AgP_6_O_21_H_11_. The isosurface value is 2*.*0 × 10^*−*2^ a.u.

We calculated the Ag(III) optical absorption and luminescence spectra for comparison with experimental spectra using DFT calculations. The computational details are available in the Materials and Methods section. First, we constructed a silver phosphate model cluster, AgP_6_O_21_ (Fig. 7a), based on the FD-7 glass model obtained using RMC. We terminated it with hydrogen atoms to avoid artificial dangling bonds (Fig. 7b). The obtained absorption spectrum based on the excited-state calculation after ground-state geometry optimization reproduced the experimentally observed peaks at wavelengths above 250 nm (Fig. 7c). The prominent peak at approximately 355 nm is ascribed to the S_19_ ← S_0_ transition. Table 3 lists the obtained excited states, whose oscillator strengths from S_0_ are relatively large, and their major electronic configurations with configuration interaction (CI) coefficients. As shown in Table 3 and Fig. 7d,e, the prominent S_19_ ← S_0_ peak mainly corresponds to the transition from the molecular orbital containing $\text {Ag-d}_{\text Z^2}$ (HOMO-18) to the molecular orbital including $\text {Ag-d}_{{\text x^2}-{\text y^2}}$ (LUMO). Therefore, the absorption is a d-d transition of Ag(III). However, it should also be noted that both of these orbitals are delocalized over Ag(III) and the phosphate ligands. In general, the oscillator strength is proportional to the square of the transition dipole moment, which increases as the overlap density between the relevant orbitals (transition density) increases. Therefore, the large absorption intensity of the S_19_ ← S_0_ transition is due to the delocalized and dipolar overlap density between HOMO-18 and the LUMO (Fig. S15). In addition, the emission wavelength of the S_19_ state was calculated to be 659 nm (Table 4), which is close to the experimentally observed emission wavelength of approximately 640 nm. Thus, the Ag(III) phosphate cluster based on the FD-7 glass model satisfactorily explained the experimentally observed absorption and luminescence spectra.

**Table 3:** Excited states of the S_0_-optimized silver(III) phosphate model AgP_6_O_21_H_11_ at the TD-B3LYP/LanL2DZ (Ag, P) & 6-31G(d, p) (O, H) level of theory.

State		Excitation energy		Oscillator strength	Major configurations (CI coefficient)
		(eV)	(nm)		
S_18_	(^1^*A*)	3.2611	380.19	0.0517	HOMO-18 → LUMO (− 0.31330)
					HOMO-17 → LUMO (0.49734)
S_19_	(^1^*A*)	3.4880	355.45	0.1615	HOMO-18* → *LUMO (0.42494)
S_20_	(^1^*A*)	3.5721	347.09	0.0502	HOMO-20* → *LUMO (0.36475)
					HOMO-19* → *LUMO (0.47946)
S_30_	(^1^*A*)	4.3500	285.02	0.0537	HOMO-28* → *LUMO (0.52485)

Major configurations with the configuration interaction (CI) coefficients are also shown. Oscillator strengths less than 0.05 are not listed.

**Table 4:** Absorption and emission vertical transition energies (VTEs) between the S_19_ and S_0_ states of the silver(III) phosphate model AgP_6_O_21_H_11_ at the TD-B3LYP/LanL2DZ (Ag, P) & 6-31G(d,p) (O, H) level of theory.

		S_19_–S_0_
Absorption VTE	(eV)	3.488
	(nm)	355
Reorganization energy	(eV)	0.803
Stokes shift	(eV)	1.607
Emission VTE	(eV)	1.881
	(nm)	659

## Discussion

In this study, we examined a silver-doped phosphate glass via combinatorial experimental techniques. We only used commercially available Ag-doped (FD-7) glass because XANES, optical absorption, and ESR are highly sensitive to the nature of the samples. For example, the signal-to-noise ratio of the XANES spectra is not sufficient at relatively low current values, and defect formation affects not only the luminescent properties but also various structural properties. Notably, different samples after XANES measurement (i.e., X-ray irradiation) were required for optical absorption, RPL, and ESR analysis because they require different sample dimensions. A previous study^15^ demonstrated that the structure and properties of samples prepared in the lab were remarkably similar but not identical to commercially available samples. Therefore, preparing the material in the lab is not sufficient to reduce the errors originating from the material fabrication process. Therefore, it is essential to examine commercially available Ag-doped bulk glass through accurate and precise analysis to obtain reproducible results for different measurement techniques.

We believed that a combinatorial measurement approach comprising Ag L-edge XANES, ESR, X-ray diffraction, neutron diffraction, Ag K-edge XAFS, spectral simulation, and DFT calculations is required for the analysis of amorphous materials, which are considerably different from periodic crystals. The use of various measurement techniques is crucial for identifying the metastable Ag species. The Ag species used as dopant in glass must be examined not only from the perspective of luminescence properties, which is the focus of this study, but also with consideration of cation mobility in glass and the conductivity of Ag cations. Therefore, the examination of trace amounts of Ag ions has been of general interest and has attracted much attention in both basic science and practical applications.

The generation of an X-ray-induced Ag^3+^ peak and RPL exhibited considerably similar saturation behaviours. In addition, the Ag^3+^ species can be erased after thermal annealing; this is a critical aspect for RPL applications. The simulated XANES spectra were in good agreement with the experimental data, confirming that the silver oxide exhibits an isoelectric configuration of Ag(I)Ag(III)O_2_. Moreover, the structure constructed via RMC modelling based on diffraction and EXFAS data yielded a plausible local coordination of Ag cations that is similar to that of Ag_2_O_3_. Furthermore, DFT calculations revealed that the absorption band originated from the coordination of Ag cations. Although a previous study confirmed the existence of Ag(III) in silver oxide thin films via X-ray photoelectron spectroscopy^43^, this study is the first to detect trace amounts of Ag^3+^ in a bulk material though XANES analysis. Although RPL is a well-studied topic, it remains scientifically relevant because the underlying mechanism is yet to be elucidated. The investigation of trace dopants in bulk materials is a challenging and attractive topic in materials science. Our findings will afford insights into the RPL behaviour of glasses and aid the future design of excellent RPL glass detectors. Furthermore, the results presented herein will immensely contribute to the future progress in the application of Ag-containing materials.

## Materials and methods

### Sample details

Commercial FD-7 glasses were provided by the Chiyoda Technol Corporation. Silver oxide clathrate (Ag_7_O_8_NO_3_) on Nb:TiO_2_ single crystals was prepared via photoelectrochemical synthesis as described in a previous study^29^. After the film preparation, the samples were stored under a vacuum in a sample container until shortly before the XANES measurements.

### Physicochemical analysis

Glass transition temperature *T*_g_ was determined at a heating rate of 10 °C/min using a differential thermal analyser (Thermo plus EVO2 Rigaku, Japan). Based on *T*_g_, an annealing process was performed using an electric hotplate in an air atmosphere.

### X-ray absorption spectroscopy (XAS) measurement

Ag L_3_-edge (3.35 keV) XAFS measurements were performed on the BL11 beamline at Kyushu Synchrotron Light Research Center (Saga, Japan). The spectra of the samples were recorded in fluorescence mode with 1- Solid State Detector at 20–25 °C using a Si (111) double-crystal monochromator. The atmosphere in the sample chamber was replaced by He gas. The XAFS data for Ag-foil (0.001 mm), AgO, and Ag_2_O were collected in the transmission mode. The XAFS data for Ag_7_O_8_NO_3_ on an Nb:TiO_2_ single crystal was collected in fluorescent mode. The duration for one L_III_-edge XANES scan was 21 min, including initialization and termination of measurement. Experimental details for Ag L_III_-edge XAFS are shown in Fig. S2–S4. Furthermore, the Ag K-edge (25.5 keV) of the XAFS spectra was measured using the BL01B1 beamline at SPring-8 (Hyogo, Japan). These measurements were performed using a Si (311) double-crystal monochromator in transmission mode (Quick Scan method). Ag foil and Ag_2_O were used as references. The corresponding analyses were performed using the Athena software^5^. The previous results on Ag K-edge XAFS are shown in a reference^15^.

### Thermal annealing

Because it has been reported that thermal annealing at 360 °C is sufficient to erase defects^28^, the reversibility of the Ag valence change was examined using the same glass sample. After the XANES measurement, the sample was removed from the sample chamber and annealed at 360 °C using a conventional heater. Thermal annealing was performed for approximately 30 min in air and the sample was then cooled to room temperature. After the sample chamber with He gas (for more than 1 h) at RT, XANES measurements were performed at the same position. The process of X-ray irradiation followed by thermal annealing was performed twice.

### High-energy XRD measurement

High-energy XRD measurements were performed on the BL04B2 beamline at SPring-8 (Hyogo, Japan) using a two-axis diffractometer dedicated to the study of disordered materials^44^. The energy of the incident X-rays was 61.34 keV. The raw data were corrected for polarization, absorption, and background, and the contribution of Compton scattering was subtracted using a standard data analysis software^44^. The corrected XRD data were normalized to obtain the total structure factor (*S*(*Q*)).

### Neutron diffraction measurement

Neutron diffraction measurements were conducted using a high-intensity total diffractometer (NOVA), installed on the BL21 beamline of the Materials and Life Science Experimental Facility at the J-PARC spallation neutron source (Ibaraki, Japan). The wavelength range of the incident neutron beam was 0.12 Å < *λ* < 8.3 Å. The glass sample (1.2 g) was transferred into a vanadium-nickel null alloy cell with an outer diameter of 6.0 mm and a thickness of 0.1 mm. The observed scattering intensity for the sample was corrected for instrumental background and attenuation of the sample and cell and then normalized by the incident beam profile. The corrected neutron diffraction data were normalized to obtain *S*(*Q*).

### Structural modelling

An atomistic model of FD-7 glass was obtained by RMC modelling^45^ using the RMC++ code^46^. The atomic number density was 0.07684 Å^–3^. The initial configuration, which contained 6000 particles, was created using a hard-sphere Monte Carlo (HSMC) simulation with constraints to avoid physically unrealistic structures. The *r*-spacing for the calculations of the partial pair-distribution functions was set to 0.05 Å. Three types of constraints were applied: the closest atom–atom approach, P–O connectivity, and Al–O connectivity. The first constraint can prevent unreasonable spikes in the partial pair-distribution functions. The second constraint forces phosphate atoms to form Q^2^ units within a P–O cutoff distance of 1.75 Å. The third constraint forces aluminium atoms to coordinate to 4–6 oxygen atoms with an AlO_4_:AlO_5_:AlO_6_ ratio obtained by NMR measurement within an Al–O cutoff distance of 2.10 Å. In addition, fixed neighbour constraints^47^ were applied for P–O and Al–O correlations at 1.40–1.75 Å and 1.70–2.10 Å to reproduce the Q^0^:Q^1^:Q^2^:Q^3^ and AlO_4_:AlO_5_:AlO_6_ ratios obtained by NMR measurements, respectively. The Q^0^:Q^1^:Q^2^:Q^3^ and AlO_4_:AlO_5_:AlO_6_ ratios obtained by RMC modelling were 0:0:100:0 and 83.21:12.86:3.93, respectively. After the HSMC simulations, the RMC simulations were conducted to reproduce the X-ray *S*(*Q*), neutron *S*(*Q*), and *k*^3^*χ*(*k*) data. Two Ag atoms exist in approximately 6000 atoms, which was calculated from the chemical composition of FD-7 glass^15^.

### XANES simulation

The Ag L-edge XANES simulation was performed using the first-principles plane-wave pseudopotential method with a generalized gradient approximation (GGA) using the CASTEP code^48^. The plane-wave cut-off energy was set to 500 eV. Because the XANES simulations required the calculation of the excited state of the core electrons, an on-the-fly pseudopotential based on CASTEP was applied to the excited Ag atoms. The theoretical excitation energy was estimated using a method reported in a previous study^36^; the ground and excited state simulations were performed separately. An error in the absolute value of the transition energy of approximately 1% of the transition energy has been reported^36^. In the present study, an error of ~ 45 eV, corresponding to approximately 1.3% of the absolute transition energy, was observed. In addition, quantitative reproduction of the chemical shift, namely relative spectrum energy, is possible using the present method^36^.

Ag-L XANES simulations for the AgF_3_, AgF_2_, Ag_2_O_3_, Ag_2_O, and AgO crystals were systematically simulated. To accurately simulate the core–hole effect, sufficiently large supercells with lengths of 9–15 Å were used in the respective directions. Because we used a large supercell, the self-consistent simulation of the electronic structure was performed by 2 × 2 × 2 k-point sampling, whereas 3 × 3 × 3 k-point sampling was used for the XANES simulations. The simulated spectra were obtained by broadening the transition probability using a Gaussian function with a standard deviation of 0.5 eV. Two types of AgO with space groups* P*2_1_/*C* and *Cccm* were simulated. AgO with space group *P*2_1_/*C* has two crystallographically different Ag sites (A and B), and the Ag L-edges of the A and B sites were simulated separately and aligned using their simulated transition energies. The A- and B-sites were 6- and fourfold sites, respectively. Because the numbers of A and B sites in the unit cell are identical, their total spectrum is their number.

The electronic structure of AgO was investigated using a hybrid functional approach and GGA + *U* approach^49^. The effects of on-site Coulomb potential and spin polarization on the simulated spectrum are shown in Fig. S16. The Ag L-edge of AgO was simulated under different conditions: no *U* (without spin), *U* = 6 eV (with spin), and *U* = 7 eV (with spin). *U* = 6 eV was reported in a previous study with results similar to those obtained using the hybrid functional approach^49^. As shown in Fig. S16, the calculation condition does not significantly affect the spectral features; therefore, simulated data without spin and no *U* were used in this study. In the simulation, the linear dichroism effect was simulated by the electron transition to the *x*, *y*, and *z* components of the wavefunctions.

In the case of Ag-L XANES simulations for Ag in FD-7 glass, we extracted a local structure around the silver atom from the RMC results for the spectral calculations. This cluster comprised 243 atoms. To simulate a trivalent silver state, we adjusted the stoichiometry of the extracted cluster.

### RPL measurement

RPL spectra were recorded at RT using an F7000 fluorescence spectrophotometer (Hitachi High-Tech, Japan). The RPL measurements were performed within 2 d after XANES measurements. Considering the fading effect of FD-7 glass^42^, the time between the XANES and RPL measurements is negligible.

### ESR measurement

X-band ESR spectra were recorded using an electron spin resonance spectrometer (JES-X330, JEOL). The microwave power, modulation field, and ESR time constants were 6 mW, 0.8 mW, and 0.03 s, respectively. All ESR measurements were carried out at − 170 °C. Samples with a width of approximately 2 mm were cut from FD-7 glass and inserted into a SiO_2_ tube for ESR measurements. The ESR measurements were performed within 2 d after XANES measurements.

### Optical absorption and luminescence calculation

For the DFT calculations of optical absorption and luminescence, a silver phosphate model cluster (AgP_6_O_21_) was constructed by extracting it from the FD-7 glass model obtained by RMC. To remove artificial dangling bonds, the cluster was terminated by hydrogen atoms so that the Ag, P, O, and H oxidation states were + 3, + 5, − 2, and + 1, respectively. Accordingly, AgP_6_O_21_H_11_ was obtained. Geometry optimization and vibrational analysis were performed at the B3LYP/LanL2dZ (Ag, P)/6-31G(d,p) (O, H) level of theory. Time-dependent DFT calculations were performed to simulate the absorption spectrum. The energy gradient was also calculated for the Franck–Condon state with a considerable oscillator strength from the ground state. All DFT calculations were performed using Gaussian 16, Revision C.01^50^. Subsequently, Stokes shifts were calculated based on the obtained energy gradient using a displaced harmonic oscillator model.

### Supplementary Information

Supplementary Information.

## Data Availability

The data that support the findings of this study are presented in the main text and the Supplementary Information, and are available from the corresponding authors upon reasonable request.
