# Supplementary material for: Combinatorial characterization of metastable luminous silver cations
Source: Sci Rep. 2024 Feb 26;14:4638. doi: 10.1038/s41598-024-55014-8 (PMC10897463; doi:10.1038/s41598-024-55014-8)
Supplement: Supplementary file 1 — Supplementary Information. [file 41598_2024_55014_MOESM1_ESM.pdf]

Supplementary materials for

Combinatorial characterization of metastable luminous silver cations

Hirokazu Masai, Masanori Koshimizu, Hiroki Kawamoto, Hiroyuki Setoyama, Yohei Onodera, Kazutaka Ikeda, Shingo Maruyama, Naoki Haruta, Tohru Sato, Yuji Matsumoto, Chika Takahashi, Teruyasu Mizoguchi

*Correspondence and requests for materials should be addressed to H.M. (e-mail: hirokazu.masai@aist.go.jp).

Contents

Fig. S1–S16

Table S1

Supplementary Note 1: Nature of FD-7 glass

The FD-7 glass, whose chemical composition is shown in Table S1, was provided by Chiyoda Technol Corporation. The glass has been used as a glass badge for personal monitoring. Figure S1 presents the physical and structural data.

Table S1.

Chemical composition and glass transition temperatures (T_g) of FD-7 glass[1].

Chemical composition (mol%)				T_g (°C)
Ag_2O	Na_2O	Al_2O_3	P_2O_5	
0.09	27.73	13.14	59.04	458

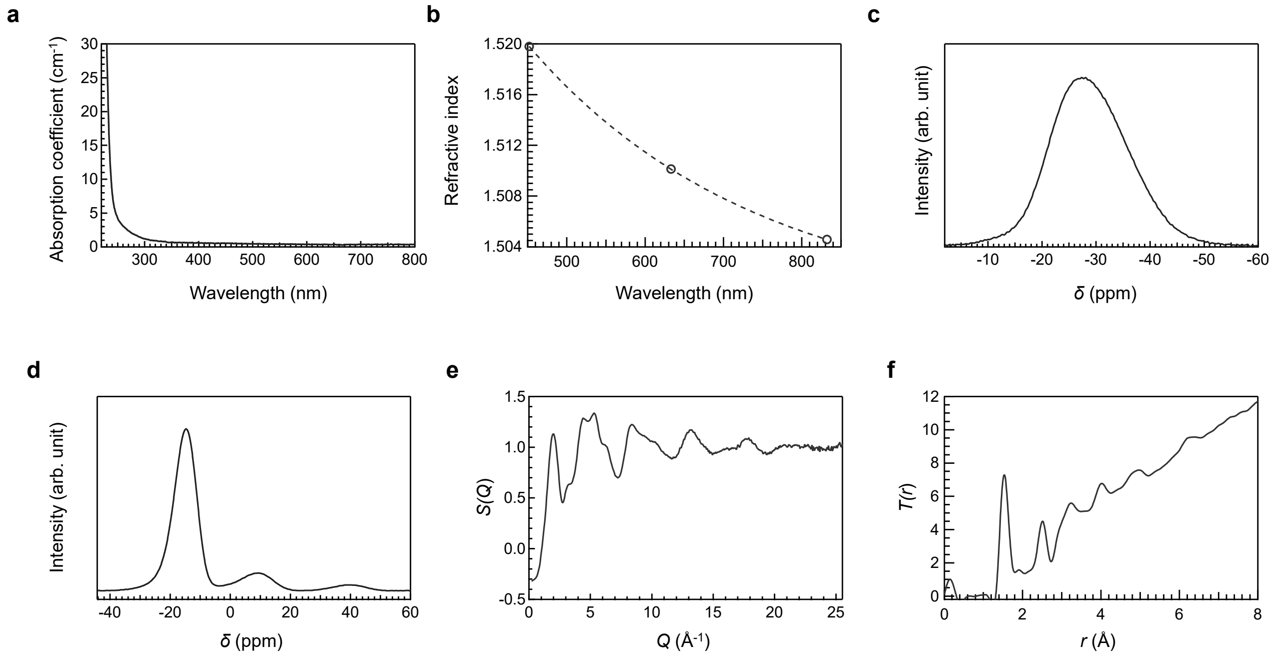

Fig. S1

Physical and structural data of FD-7 glass. (a) Optical absorption spectrum, **(b)** refractive index, **(c)** ^{27}Al MAS NMR spectrum, **(d)** ^{31}P MAS NMR spectrum, **(e)** X-ray total structure factor $S(Q)$, and **(f)** X-ray total correlation function $T(r)$ of FD-7 glass[1].

Supplementary Note 2: Measurement of Ag- L_3-edge XANES spectra

Figures S2–S4 show the conditions under which experiments were conducted at the Kyushu Synchrotron Light Research Center.

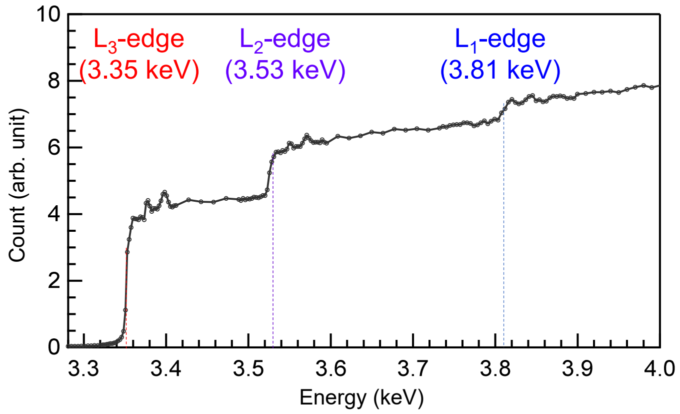

Fig. S2

Comparison of L-edge XAFS spectra of Ag foil. The L_3-edge derived from the $2p_{3/2}$→$4d_{5/2}$ transition has the largest absorption due to the dipole-allowed transition.

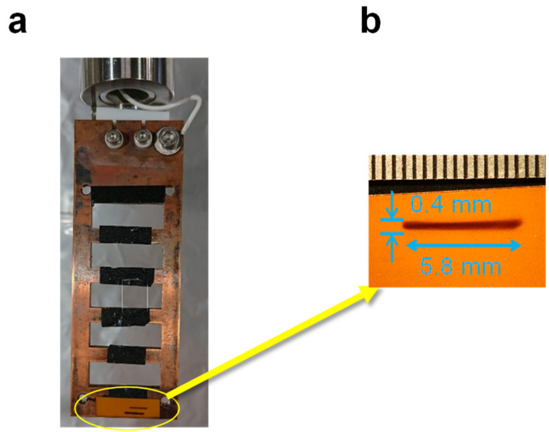

Fig. S3

Defect formation in FD-7 glass by X-ray irradiation. (a) Photograph of the sample holder for XANES measurement. **(b)** Optical micrograph of the GAFCHROMIC film after X-ray irradiation. From the image, the beam size for the XANES measurement is estimated to be 0.4 × 5.8 mm^2, which is too small to measure the irradiated part of the optical absorption spectrum.

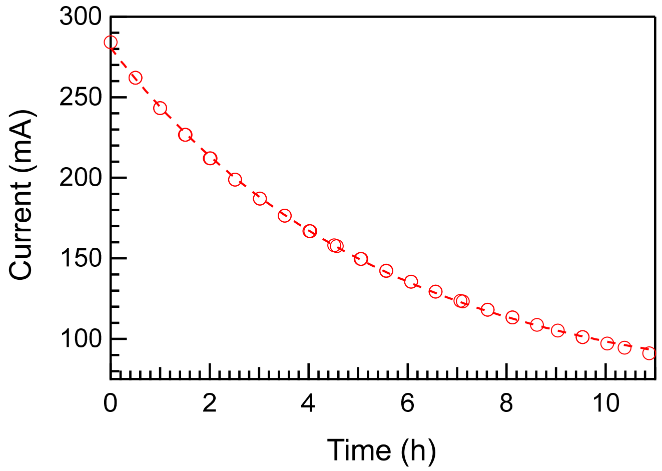

Fig. S4

Decay of electrical current during irradiation for 1 d at SAGA-LS. From the decay curve, we evaluated the average current per day to be 170 mA·h.

Supplementary Note 3: X-ray-induced change in FD-7 glass

The X-ray-induced change in the FD-7 glass increased with increasing irradiation dose and then appeared to saturate after a long irradiation time (Fig. S5). These changes were detected by optical absorption (Fig. S6), XANES (Fig. S7), and ESR (Fig. S8) measurements.

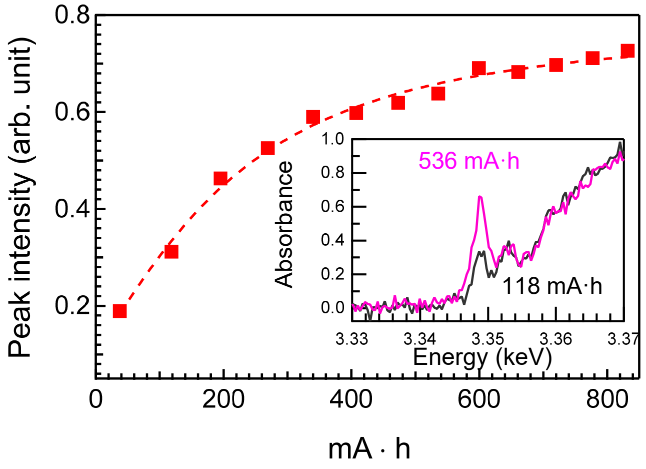

Fig. S5

Comparison of Ag L_3-XANES spectra. Generated absorption peak intensity at 3.349 keV for commercial FD-7 glass as a function of electrical current multiplied by time. Inset: Ag L_3-edge XANES spectra of FD-7 glass after different X-ray irradiation doses (118 and 536 mA·h).

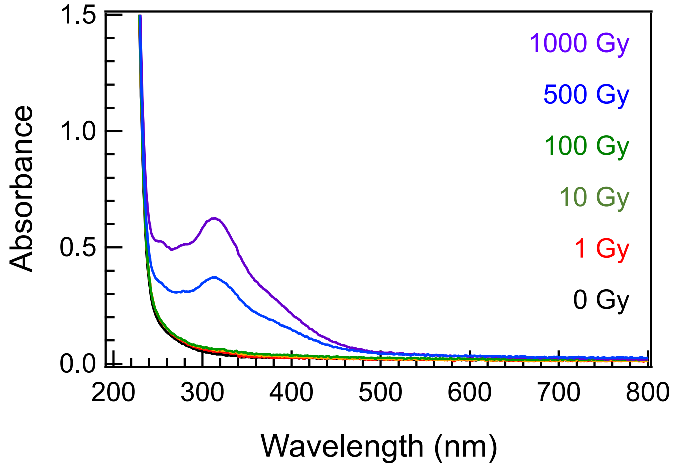

Fig. S6

Representative optical absorption spectra of FD-7 glass after different X-ray irradiation doses.

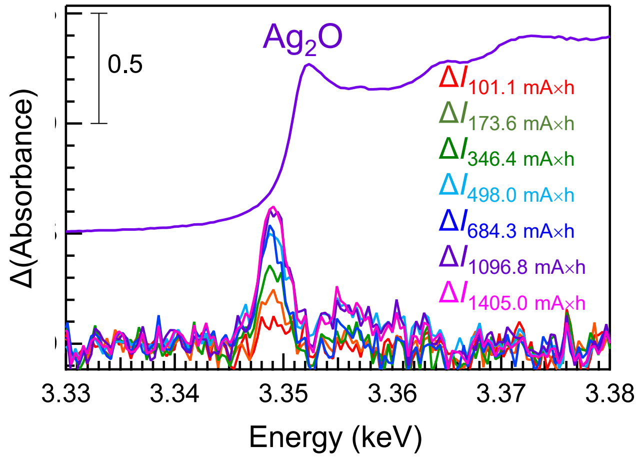

Fig. S7

Change in absorption as a function of irradiation dose. Differential XANES spectra of FD-7 glass obtained by subtracting the absorbance of FD-7 glass after 46.4 mA·h of irradiation from that of the other FD-7 glass sample. The absorption spectrum of Ag_2O is shown for comparison.

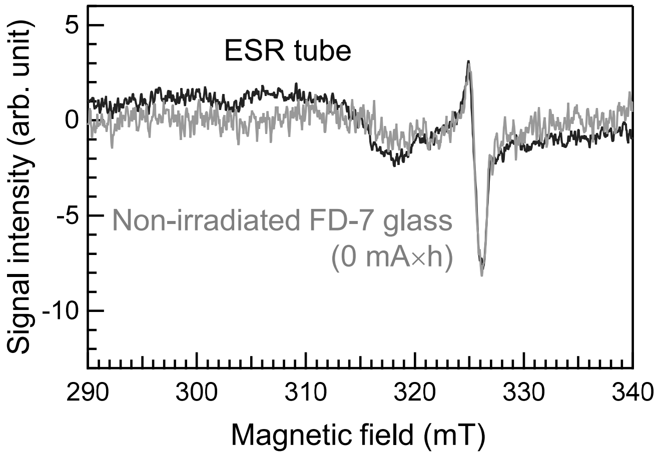

Fig. S8

Assignment of peak observed at 325 mT in ESR measurement. ESR spectra of a silica tube used for ESR measurements and non-irradiated FD-7 glass in the silica tube. The peak at g = 2.007 is assigned as the oxygen hole centre for SiO_2.

Supplementary Note 4: Structural modelling of FD-7 glass by RMC modelling

Recently, we reported on the atomic configuration of oxide glasses derived from reverse Monte Carlo (RMC) modelling based on a combination of ^{31}P MAS NMR, Zn K-edge extended EXAFS, and X-ray and neutron diffraction data[2–4]. Several datasets from different measurement techniques are used as constraints and are essential in modelling reliable structures. A reliable structural model of FD-7 was created using this approach.

As the raw data of Ag K-edge EXAFS were not suitable for fitting because of the noise of the signal derived from the low concentration, back-Fourier transformed data were used (Fig. S9). Figure S10 presents the cation–oxygen coordination number distributions in the FD-7 glass and the partial pair correlation functions $g_{ij}(r)$. Only two Ag cations were present in the RMC model containing 6,000 atoms. Ag coordination in the FD-7 glasses is illustrated in Fig. S11 (and another is shown in Fig. 5e).

RMC modelling is affected by coordination number constraints. Because two sets of oxygen coordination are forced on the Ag cations, which correspond to the local structure in Ag_2O, the reliability of the RMC-generated atomistic model can be validated. We compared the experimental data with three RMC models: (1) an RMC model without an Ag–O constraint, (2) an RMC model with a constraint of $N_{Ag–O} = 2$, and (3) an RMC model without EXAFS fitting. As Fig. S12 shows, the RMC modelling without the constraint of Ag–O coordination was most consistent with the experimental EXAFS oscillation data.

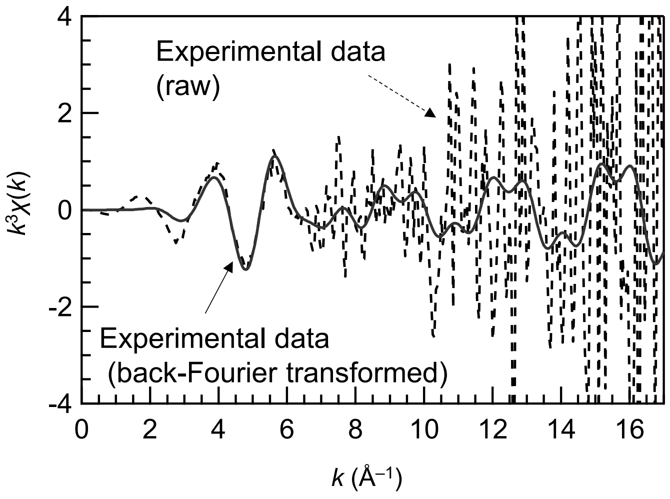

Fig. S9

Comparison of Ag K-edge EXAFS data of FD-7 glass. Raw data (solid line) and back-Fourier transformed data (dotted line).

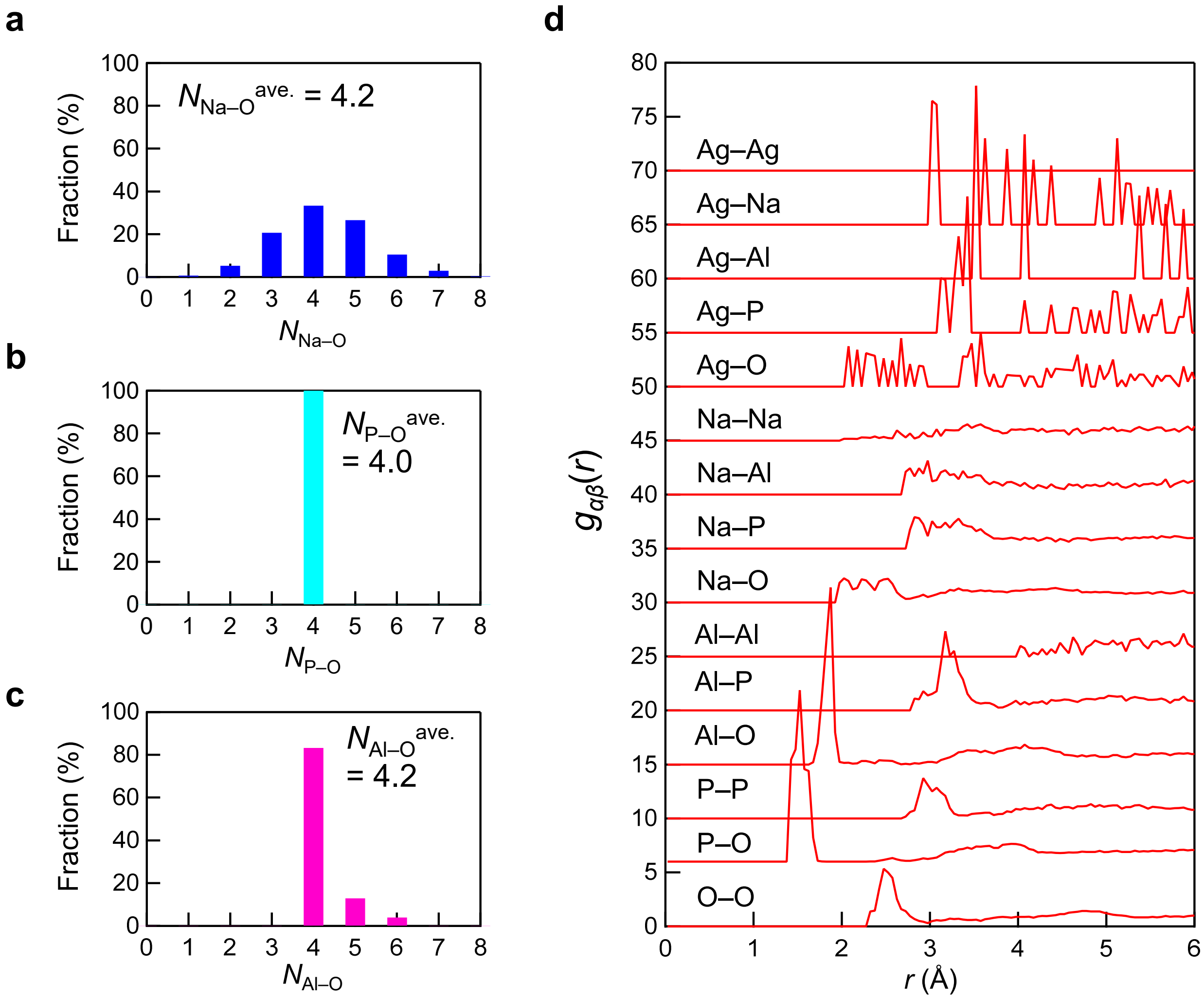

Fig. S10

RMC-generated structural parameters of FD-7 glass. Oxygen–cation coordination number distributions of: **(a)** Na, **(b)** P, and **(c)** Al in FD-7 glass. **(d)** RMC-generated partial-pair correlation functions $g_{ij}(r)$.

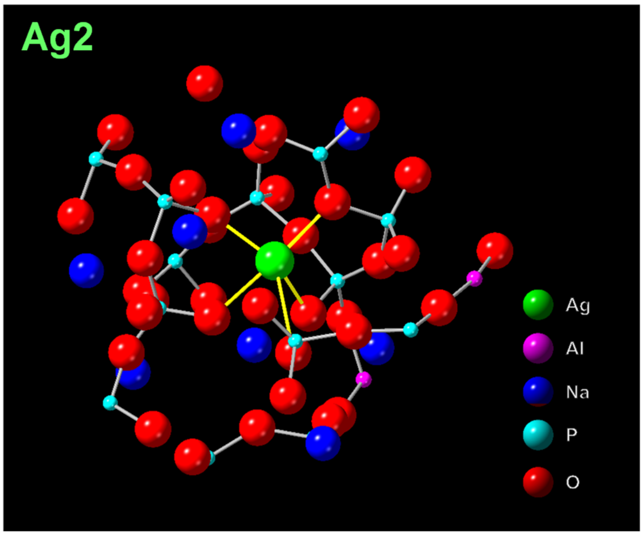

Fig. S11

Network structure of FD-7 glass obtained from RMC modelling. Ag coordination in FD-7 glasses. From the chemical composition, atomic ratios of P:Na:Al:Ag:O = 59.04:27.73:13.14:0.09:209 to 656:308:146:1: 2,320[1]. In the RMC model using 6,000 atoms, only two Ag cations are present, namely, the Ag shown in this figure and that in Fig. 5e. The P=O bonds are expected to capture electrons to compensate for the charge valance when the valence state of Ag^{+} cations is increased.

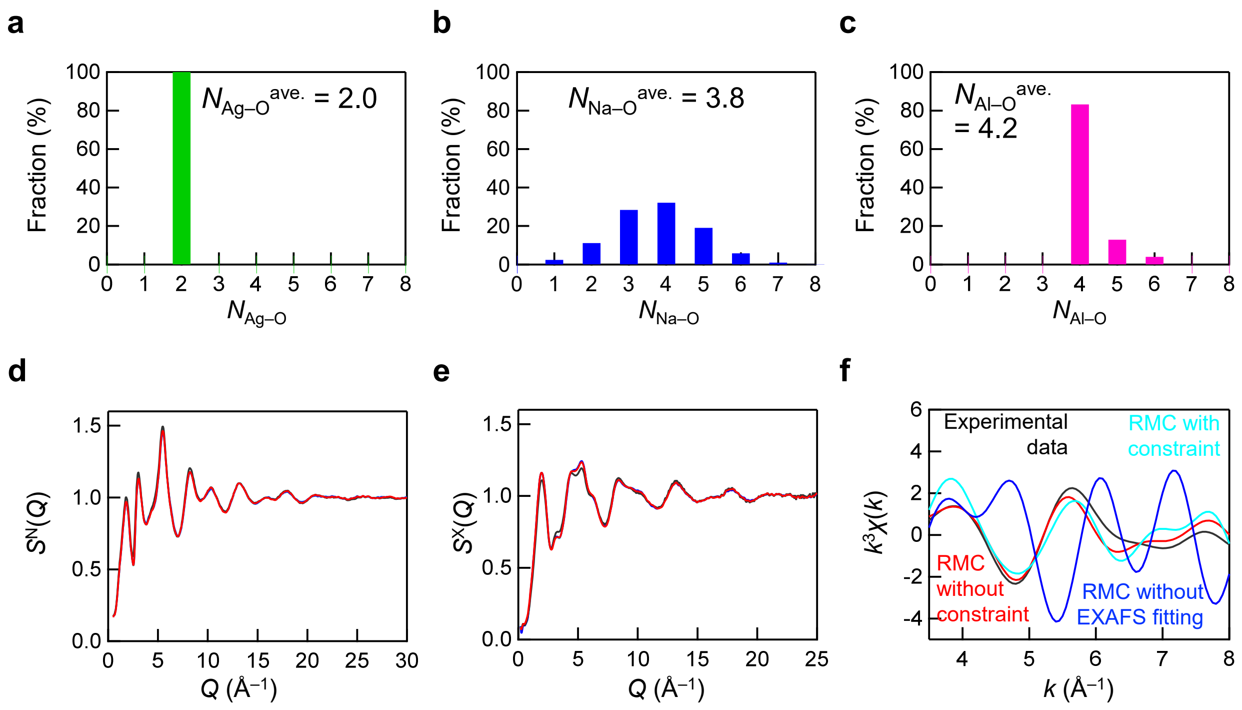

Fig. S12

Coordination numbers and comparison between neutron/X-ray data and RMC models for DF-7 glass with/without Ag coordination constraint. The coordination number distribution of oxygen around **(a)** Ag, **(b)** Na, and **(c)** Al in the RMC model with a structural constraint for Ag_2O coordination ($N_{Ag–O}$ = 2). **(d)** Neutron total structure factor $S^N(Q)$, (**e**) X-ray total structure factor $S^X(Q)$, and (**f**) Ag K-edge EXAFS spectra of FD-7 glass with and without the structural constraint. Black, red, cyan, and blue lines show the experimental data and the RMC models without an Ag–O constraint, with a constraint of $N_{Ag–O}$ = 2, and without EXAFS fitting, respectively.

Supplementary Note 5: Valence estimation of silver in FD-7 glass

The anisotropy of the L_3-edge in the $2p_x$, $2p_y$, and $2p_z$ directions must be considered in XANES spectral simulations. Figure S13 shows the detailed intensities in each direction for several Ag compounds. The shape of the experimental AgO spectrum could not be reproduced using AgO with space group *Cccm*. In the XANES spectra, the de-occupation of the states as derived from the Ag 4d orbitals was clearly observed in the experimental and simulated results. This increase in the white-line intensity was more evident in silver fluoride, as shown in Fig. S14.

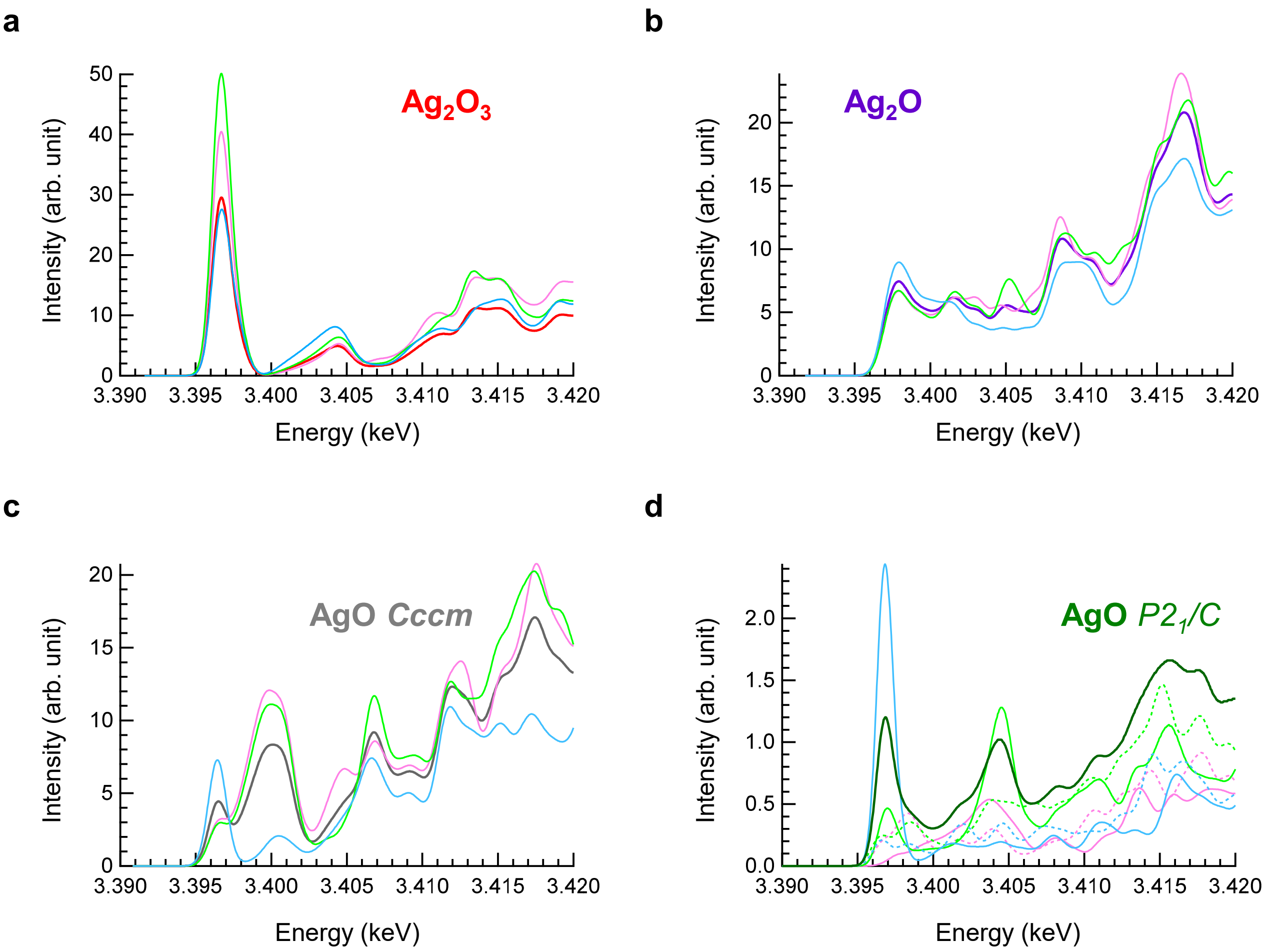

Fig. S13

Directional dependence of simulated XANES spectra of various Ag species. Average values of **(a)** Ag_2O_3, **(b)** Ag_2O, **(c)** AgO with space group *Cccm*, and **(d)** AgO with space group $P2_1/c$ are depicted in bold lines. The x, y, and z directional components are given as pink, light-green, and sky-blue lines, respectively. Directional dependence can be observed using a linear dichroism observation of a single

crystal. In AgO with space group $P2_1/c$, contributions of the A site (6-fold) and B site (4-fold) are shown as solid and dotted lines, respectively.

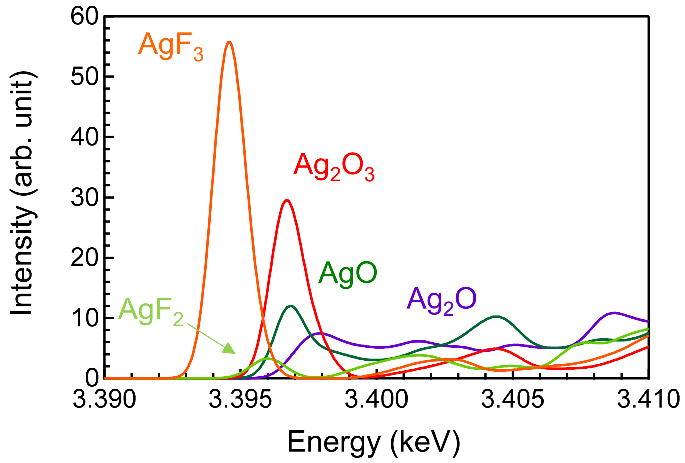

Fig. S14

Comparison of the simulated XANES spectra of Ag oxides and Ag fluorides. The absorption energy E_0 values of Ag fluorides are less than those of Ag oxides. The white-line intensities of Ag^{3+} are higher in both oxides and fluorides.

Supplementary Note 6: DFT calculation of the Ag(III) centre in FD-7 glass

The oscillator strength is proportional to the square of the transition dipole moment, which increases with the overlap density between the relevant orbitals. Figure S15 shows the overlapping densities of molecular orbitals of $AgP_6O_{21}H_{11}$. The large absorption intensity of the S_{19}←S_0 transition derives from the delocalised and dipolar overlap density between HOMO-18 and the LUMO.

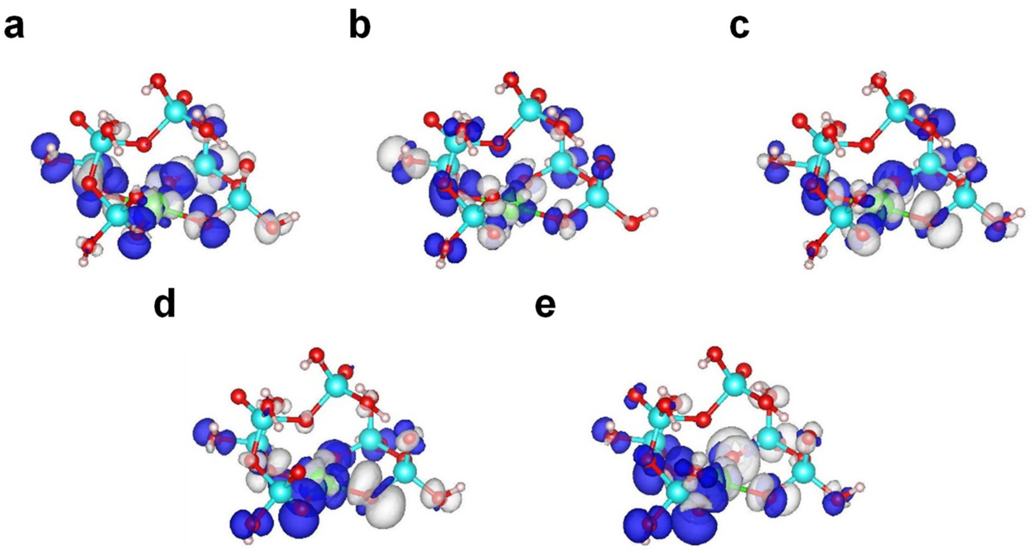

Fig. S15

Overlap densities of molecular orbitals of $AgP_6O_{21}H_{11}$. (a) HOMO-28→LUMO, **(b)** HOMO-20 →LUMO, **(c)** HOMO-19→LUMO, **(d)** HOMO-18→LUMO, and **(e)** HOMO-17→LUMO; these orbital transitions are the main components of the S_{18}←S_0, S_{19}←S_0, S_{20}←S_0, and S_{30}←S_0 transitions, respectively. The TD-B3LYP/LanL2DZ (Ag, P) & 6-31G(d,p) (O, H) level of theory was employed. The isosurface value is 4.0×10^{-4} a.u.

Supplementary Note 7: Validity of XANES simulation of AgO

Watson et al. showed that GGA density functionals cannot accurately describe the electronic structure of AgO[5]. The GGA functionals in CASTEP used to simulate the XANES spectra may result in unreliable predictions. Therefore, the electronic structure of AgO was investigated using both hybrid functional and GGA + U approaches[6]. Figure S13 shows the effects of the onsite Coulomb potential and spin polarisation on the simulated spectrum. The Ag L-edge of the AgO was simulated under the following conditions: no U (without spin), $U = 6$ eV (with spin), and $U = 7$ eV (with spin). $U = 6$ eV was reported in a previous study with results similar to those obtained using the hybrid functional approach[6]. As Fig. S16 shows, the calculation conditions did not significantly affect the spectral features; thus, simulated data without spin (no U) were used in this study. The linear dichroism effect was simulated by the electron transition to the x, y, and z components of the wave functions.

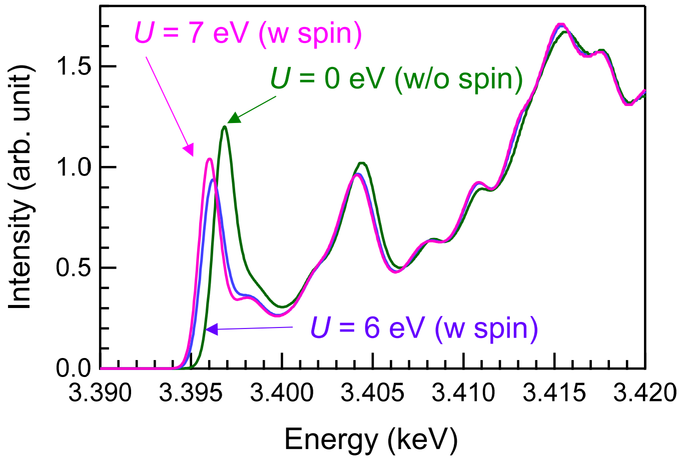

Fig. S16

Dependence of the simulated XANES spectra of AgO on the simulation conditions. The electronic structure of AgO has previously been investigated using hybrid functional and GGA + U approaches, and $U = 6$ eV has been reported to yield similar results as those under the hybrid functional approach[6]. The Ag L edge was simulated under different conditions, including $U = 0$ (without spin), $U = 6$ eV (with spin), and U =7 eV (with spin). The calculation conditions mostly did not affect the spectral features, and thus the simulated data without spin (no U) was used in this study.

Supplementary References

1. Masai, H. et al. X-ray absorption near-edge structure of Ag cations in phosphate glasses for radiophotoluminescence applications. *J. Ceram. Soc. Jpn* **127,** 924–930 (2019).
2. Onodera Y. et al. Formation of metallic cation-oxygen network for anomalous thermal expansion coefficients in binary phosphate glass. *Nat. Commun.* **8,** 15449 (2017).
3. Onodera Y. et al. Origin of the mixed alkali effect in silicate glass. *NPG Asia Mater.* **11,** 75 (2019).
4. Masai, H. et al. Correlation between structures and physical properties of binary $ZnO–P_2O_5$ glasses. *Phys. Status Solidi B* **257,** 2000186 (2020).
5. Allen, J. P., Scanlon, D. O. & Watson, G. W. Electronic structure of mixed-valence silver oxide AgO from hybrid density-functional theory. *Phys. Rev. B* **81,** 161103(R) (2010).
6. Allen, J. P., Scanlon, D. O. & Watson, G. W. Electronic structures of silver oxides. *Phys. Rev. B*, **84,** 115141 (2011).
